# A dominance hypothesis argument for historical genetic gains and the fixation of heterosis in octoploid strawberry

**DOI:** 10.1093/genetics/iyae159

**Published:** 2024-10-10

**Authors:** Mitchell J Feldmann, Dominique D A Pincot, Danelle K Seymour, Randi A Famula, Nicolás P Jiménez, Cindy M López, Glenn S Cole, Steven J Knapp

**Affiliations:** Department of Plant Sciences, University of California, One Shields Avenue, Davis, CA 95616, USA; Department of Plant Sciences, University of California, One Shields Avenue, Davis, CA 95616, USA; Department of Botany and Plant Sciences, University of California, 900 University Avenue, Riverside, CA 92521, USA; Department of Plant Sciences, University of California, One Shields Avenue, Davis, CA 95616, USA; Department of Plant Sciences, University of California, One Shields Avenue, Davis, CA 95616, USA; Department of Plant Sciences, University of California, One Shields Avenue, Davis, CA 95616, USA; Department of Plant Sciences, University of California, One Shields Avenue, Davis, CA 95616, USA; Department of Plant Sciences, University of California, One Shields Avenue, Davis, CA 95616, USA

**Keywords:** hybrid vigor, directional dominance, inbreeding depression, polyploid, *Fragaria*, Plant Genetics and Genomics

## Abstract

Heterosis was the catalyst for the domestication of cultivated strawberry (Fragaria×ananassa), an interspecific hybrid species that originated in the 1700s. The hybrid origin was discovered because the phenotypes of spontaneous hybrids transgressed those of their parent species. The transgressions included fruit yield increases and other genetic gains in the twentieth century that sparked the global expansion of strawberry production. The importance of heterosis to the agricultural success of the hybrid species, however, has remained a mystery. Here we show that heterosis has disappeared (become fixed) among improved hybrids within a population (the California population) that has been under long-term selection for increased fruit yield, weight, and firmness. We found that the highest yielding hybrids are among the most highly inbred (59–79%), which seems counterintuitive for a highly heterozygous, outbreeder carrying heavy genetic loads. Although faint remnants of heterosis were discovered, the between-parent allele frequency differences and dispersed favorable dominant alleles necessary for heterosis have decreased nearly genome-wide within the California population. Conversely, heterosis was prevalent and significant among wide hybrids, especially for fruit count, a significant driver of genetic gains for fruit yield. We attributed the disappearance (fixation) of heterosis within the California population to increased homozygosity of favorable dominant alleles and inbreeding associated with selection, random genetic drift, and selective sweeps. Despite historical inbreeding, the highest yielding hybrids reported to-date are estimated to be heterozygous for 20,370–44,280 of 97,000–108,000 genes in the octoploid genome, the equivalent of an entire diploid genome or more.

## Introduction

The domestication of plants and animals in the Holocene Epoch (approximately 9,000 BCE to present) profoundly changed global demography, created the staple foods that dominate modern human diets, and expanded human exploration and migration (Diamond 2002). The increased mobility of human populations from the start of the Columbian Exchange (1,492 CE) onward accelerated the discovery and exchange of food plants between the New and Old Worlds (Crosby 2003; Nunn and Qian 2010), including the self- and cross-compatible progenitors of the hybrid species known around the world today as cultivated strawberry (Fragaria×ananassa Duchesne; Duchesne 1766; Darrow 1966). Among the intercontinental migrants were two nonsympatric New World octoploid (2n=8x=56) strawberry taxa (*F. chiloensis* subsp. *chiloensis* and *F. virginiana* subsp. *virginiana*) that spontaneously hybridized in Old World gardens in the early 1700s, the origin of F.×ananassa domestication (Duchesne 1766; Darrow 1966). The discovery of those original chance hybrids and subsequent breeding ultimately transformed strawberry from a seasonally produced fruit with limited production to one of the most widely produced and consumed fruits in the world (Feldmann *et al*. 2024; https://www.fao.org/food-agriculture-statistics/en/).

The conscious domestication of F.×ananassa began soon after Duchesne (1766) deduced that individuals with increased vigor and transgressive phenotypes were offspring of spontaneous hybrids between *F. chiloensis* and *F. virginiana*, a scientific discovery far ahead of its time (Staudt 2003; Ratcliff 2007). The transgressive phenotypes and heterosis of those early spontaneous hybrids were catalysts for the domestication of the artificial hybrid species, although heterosis was curiously only briefly mentioned in the definitive narrative history of strawberry domestication (Darrow 1966), and then only superficially for early F.chiloensis×F.virginiana hybrids and hybrids developed by Albert F. Etter at the turn of the twentieth century between F.×ananassa cultivars and native California *F. chiloensis* ecotypes (Clausen 1915). Hybrids that mimic the latter were developed for our study.

East (1936), one of the pioneers of the study of heterosis, observed that strawberry and other “asexually propagated horticultural plants derive the vigor that has made them useful from heterosis, since they invariably lose much of this vigor when inbred”, and further that strawberry “species belonging to different subgenera show more heterosis than hybrids between species belonging to the same subgenera”, ironically without reference to the artificial homoploid hybrid species (F.×ananassa), which by then dominated strawberry cultivation and breeding nearly everywhere (Darrow 1966; Hancock *et al*. 2008; Finn *et al*. 2013). Despite the present-day obviousness of those conclusions, heterosis did not become a guiding principle in plant breeding until the phenomenon was “rediscovered” by Shull (1908) and East and Jones (1919) in the early twentieth century and fundamentally transformed maize breeding practices (Lamkey and Edwards 1999; Duvick 2001; Kaeppler 2012; Schnable and Springer 2013), but does not appear to have ever been a guiding principle in the breeding practices widely applied in strawberry (Darrow 1966; Shaw 1995, 1997; Whitaker *et al*. 2012; Pincot *et al*. 2021; Feldmann *et al*. 2024).

Using generation-means theory for a single locus with two alleles and different mating designs, Lamkey and Edwards (1999) showed that “heterosis is dependent on directional dominance”, “a function of the square of the difference in allelic frequency between two populations”, and “specific to a particular cross”. These are the core elements of the “dominance hypothesis” of heterosis (Crow 1998; Birchler *et al*. 2003, 2010; Schnable and Springer 2013). Although heterosis cannot be reduced to a single, overly simplistic genetic mechanism, the importance of directional dominance and complementation (the “dominance hypothesis”) are strongly supported by empirical studies in domesticated plants (Birchler *et al*. 2003, 2010; Kaeppler 2012; Schnable and Springer 2013; Labroo *et al*. 2021). As Schnable and Springer (2013) recounted, “an important prediction of the dominance hypothesis is that, given sufficient recombination and selection, it should be possible to generate from a hybrid an inbred that contains all of the favorable alleles and that therefore has a performance equal to that of the parental hybrid”. This has not been achieved or reported in maize or any other heterotic plant species, presumably because of historical between-population breeding practices and the challenge of eliminating deleterious recessive alleles (Schnable and Springer 2013; Yang *et al*. 2017). Our hypothesis is that directional dominance has been critically important to the historical genetic gains for heterotic traits in strawberry, and that heterosis has become increasingly less detectable (decreased in frequency and magnitude) in populations where favorable dominant alleles have been driven to fixation or near fixation by artificial selection and inbreeding (Feldmann *et al*. 2024). We empirically explore this dominance hypothesis prediction in a specific population (the California population); however, the breeding history of strawberry suggests that the allele frequency differences necessary for detecting heterosis have decreased and possibly disappeared in other partially to completely closed populations under long-term selection for domestication traits (Hardigan *et al*. 2021; Pincot *et al*. 2021).

The descendants of the interspecific founders of cultivated strawberry have been recurrently interbred, admixed, and inbred through the application of within-family and -population breeding schemes where the unit of selection has been the hybrid individual (Darrow 1966; Hancock *et al*. 2008; Gil-Ariza *et al*. 2009; Horvath *et al*. 2011; Whitaker *et al*. 2012; Sánchez-Sevilla *et al*. 2015; Vallarino *et al*. 2018; Pincot *et al*. 2021). As a consequence of breeding bottlenecks and artificial selection, effective selfing rates have progressively increased in many strawberry populations (Ritland 1984; Hardigan *et al*. 2021), especially those that have been closed for several generations, and where genetic gains from long-term selection have been significant, e.g. the “California” population that has emerged at the University of California, Davis over the last 70 years (Bringhurst and Voth 1980, 1984; Shaw and Larson 2008; Feldmann *et al*. 2024). That population has been an important source of elite genetics and groundbreaking cultivars (asexually propagated hybrid individuals), played a significant role in the expansion of strawberry production, and was the source of the elite parents of hybrids created for the studies reported here (Bringhurst and Voth 1980, 1984; Sjulin 2003; Hancock 2006; Sjulin 2006; Shaw and Larson 2008; Feldmann *et al*. 2024).

Although inbreeding depression and heterosis have been reported in strawberry (East 1934, 1936; Shaw 1995, 1997; Murti *et al*. 2012; Rho *et al*. 2012; Kaczmarska *et al*. 2016, 2017; Kaczmarska and Gawronski 2019), the importance of heterosis to genetic gains for yield and other domestication traits are unclear (Feldmann *et al*. 2024). Heterosis could theoretically be caused by a combination of between-group and inter-subgenomic heterosis in this interspecific hybrid species (East 1934, 1936; Comai 2005; Birchler *et al*. 2010; Washburn and Birchler 2014). The latter has been described as “progressive” heterosis, a phenomenon attributed to an increase in gene dosage and interactions between redundant genes in the ancestral subgenomes of polyploids (Birchler *et al*. 2010; Washburn and Birchler 2014). While the importance of progressive heterosis in the octoploid progenitor species is unknown, between-group heterosis and transgressive segregation of dispersed favorable alleles were catalysts for the domestication of the hybrid species as opposed to the parent species *per se* (Duchesne 1766; Clausen 1915; East 1934; Darrow 1966; Hancock *et al*. 2010; Finn *et al*. 2013). The breeding history of strawberry clearly shows that between-group heterosis has not been formally exploited, at least not beyond the ancestral interspecific heterotic pattern (*F. chiloensis* × *F. virginiana*) that was the driving force behind the domestication of the hybrid species in the first place (Duchesne 1766; Darrow 1966).

Our study explores the interconnections between historical inbreeding and genetic gains, genome-wide reshaping of allelic variation, and greatly improved hybrid performance using modern descendants from a unique population (the California population) with a long selection history (Hardigan *et al*. 2021; Pincot *et al*. 2021; Feldmann *et al*. 2024). The nucleotide diversity found in the genomes of that population and other domesticated strawberry populations remained a mystery until the first highly contiguous octoploid genome was assembled using DNA of the cultivar “Camarosa” (Edger *et al*. 2019). That genome assembly, while imperfect and unphased, marked an important milestone in octoploid strawberry breeding and genetics and supplied the subgenome-resolved framework needed for genome-wide analyses of DNA variants in the octoploid species (Edger *et al*. 2019; Hardigan *et al*. 2020, 2021). Those analyses uncovered massive nucleotide diversity and heterozygosity in the genomes of modern cultivars (Hardigan *et al*. 2021) and challenged the prevailing belief that *F.* × *ananassa* is “genetically narrow” and more specifically that genetic variation for improving strawberry has been inadequate (Sjulin and Dale 1987; Dale and Sjulin 1990; Hancock and Luby 1993; Hancock *et al*. 2001, 2008, 2010; Stegmeir *et al*. 2010; Gaston *et al*. 2020). Despite the logic behind those beliefs, genome-wide analyses of DNA sequence variation suggest that substantial genetic variation exists in domesticated strawberry populations (Hardigan *et al*. 2021), partly in the form of unfavorable, incompletely dominant alleles that have not yet been purged by breeding and diminish hybrid performance when exposed by inbreeding (Gore *et al*. 2009; Yang *et al*. 2017; Hardigan *et al*. 2021; Lozano *et al*. 2021; Feldmann *et al*. 2024). We have argued that historical genetic gains from breeding for yield and other domestication traits, which have been substantial, could not have been achieved without significant genetic variation, despite breeding bottlenecks, increased inbreeding (declines in nucleotide diversity and heterozygosity), and genetic erosion (Hill *et al*. 2008; Whitaker *et al*. 2012; Feldmann *et al*. 2024).

Here we show that hybrids between parents developed by long-term selection for increased fruit yield, weight, and firmness within the California population, despite being moderately to highly inbred, are exceptionally high yielding and segregate (heterozygous) for the equivalent of a typical diploid genome or more (Michael and Jackson 2013; Kersey 2019; Hardigan *et al*. 2021; Feldmann *et al*. 2024). The latter was informed by analyses of haplotype-phased assemblies of the genome of the high yielding hybrid “Royal Royce” (https://phytozome-next.jgi.doe.gov/info/FxananassaRoyalRoyce_v1_0), and by previous genome-wide analyses of nucleotide diversity and heterozygosity and population structure (Hardigan *et al*. 2021). We present a “dominance hypothesis” argument to explain historical genetic gains and the apparent fixation of heterosis within the California population and show that the improved performance of California population hybrids has been achieved by within-population selection whilst avoiding inbreeding depression (Lamkey and Edwards 1999; Birchler *et al*. 2003, 2010; Kaeppler 2012; Schnable and Springer 2013). We hypothesize that heterosis, which appears to have been prevalent and substantial in early founders of the California population, disappeared in modern descendants as they progressively accumulated incompletely to completely dominant favorable alleles nearly genome-wide. Our findings suggest that the allele frequency differences and dispersed favorable dominant alleles necessary for hybrid vigor (heterosis) have largely been eliminated by selection and inbreeding within the California population (Falconer and Mackay 1996; Lamkey and Edwards 1999; Kaeppler 2012; Mackay *et al*. 2021). The implications of our findings are discussed in the context of hybrid breeding strategies in strawberry and other asexually propagated hybrid plants where inbred-hybrid breeding schemes are either impractical or unnecessary for maximizing hybrid performance (Labroo *et al*. 2021).

## Materials and methods

### Plant material and mating design

We developed 75 F.×ananassa full-sib families using an incomplete factorial (14×16) mating design with 27 elite and three exotic parents (Supplementary File 1). The female parents were hand emasculated for hybrid seed development. We grew 2,800 hybrid individuals (full-sib progeny) from the 75 of those families to physioloigcal maturity at the Wolfskill Experiment Orchard in 2016–2017. We randomly selected 356 elite×elite and 113 elite × exotic hybrids across full-sib families for clonal propagation and inclusion in field studies for phenotyping (Supplementary File 1).

Of the 27 elite parents, 13 were classified as photoperiod sensitive (short-day flowering) and 14 as photoperiod insensitive (day-neutral flowering). Their photoperiod sensitivities were verified through three years of field observation. They originated between 1988 and 2011 in the University of California, Davis (UCD) Strawberry Breeding Program and included several historically and commercially important cultivars (asexually propagated hybrid individuals) publicly released by the University of California, Davis (Supplementary File 1).

The three exotic parents (“Puget Reliance”, “Oso Flaco”, and “Del Norte”) are photoperiod sensitive. “Puget Reliance” (PI664321) is an F.×ananassa cultivar released in 1994 for fresh market and processing production in the Pacific Northwest (USPP9310P). “Oso Flaco” (55C023P001) is a hybrid developed in 1955 between the heirloom F.×ananassa cultivar “Lassen” (36C003P001 developed in 1936) and an extinct *F. chiloensis* subsp. *lucida* ecotype originally collected from the Guadalupe-Nipomo Dunes near Oso Flaco Lake, Guadalupe, California by Royce S. Bringhurst. “Del Norte” (PI551753) is an *F. chiloensis* subsp. *lucida* ecotype originally collected from coastal Washington. We developed and phenotyped 52 elite × “Del Norte”, 14 elite × “Oso Flaco”, and 47 elite × “Puget Reliance” hybrids.

“Oso Flaco” and the other UC Davis individuals identified by 10-digit identification numbers in a year-family-individual format (e.g. 55C023P001) are clonally preserved in the UC Davis Strawberry Germplasm Collection (Supplementary File 1). “Puget Reliance” and “Del Norte”, the individuals identified by plant introduction (PI) numbers, were initially acquired as bare-root plants from the United States Department of Agriculture (USDA) National Plant Germplasm System (NPGS) National Clonal Germplasm Repository, Corvallis, Oregon, USA (https://www.ars.usda.gov/pacific-west-area/corvallis-or/national-clonal-germplasm-repository/). They were subsequently increased by asexual propagation at Wolfskill Experiment Orchard and incorporated into the UC Davis Strawberry Germplasm Collection (see Supplementary File 1).

Of the 30 parents and 469 hybrids included in our study, 48% originated from elite day-neutral × elite day-neutral, 25% originated from elite day-neutral × elite short-day, and 22% originated from elite day-neutral × exotic short-day crosses. We used a SNP marker on the 50K Axiom array (AX-184937335) found to be in linkage disequilibrium with the *PERPETUAL FLOWERING* (*PF*) locus to ascertain *PF* locus genotypes (Supplementary File 2; Bringhurst and Voth 1980; Bringhurst *et al*. 1989; Ahmadi *et al*. 1990). The day-neutral parents were predicted to be heterozygous for the dominant allele (*PF*), whereas the short-day parents were predicted to be homozygous for the recessive allele (*pf*). *PF*-associated SNP genotypes are documented in Supplementary File 1. The day-neutral parents were heterozygous, whereas the short-day parents were homozygous for the recessive allele; hence, full-sib families developed from elite day-neutral × elite day-neutral crosses were expected to segregate 3 *PF*_ : 1 *pfpf*, whereas full-sib families developed from elite day-neutral × elite short-day and elite day-neutral × exotic short-day crosses were expected to segregate 1 *PFpf* : 1 *pfpf*. Using the *PF*-associated SNP genotypes to classify hybrids, 171 were predicted to be photoperiod sensitive (*pfpf*), whereas 298 were predicted to be photoperiod insensitive (*PFPF* or *Pfpf*).

### Experiment design

Twenty-nine of the 30 parents and 469 hybrids were grown on a commercial farm in Salinas, CA ($36.62^{\circ }$ N, $-121.54^{\circ }$ W, 46 m) over the 2017–2018 and 2018–2019 growing seasons. The “Oso Flaco” parent could not be clonally propagated and therefore could not be included in the field experiment. The field was preplant flat-fumigated with a chloropicrin-based fumigant (Pic-Clor 60; 560 kg/ha) and sealed with an impermeable film tarp for one-week post-fumigation. Once the tarps were removed, fields were prepared for planting by pulling 30.0 cm high × 75.0 cm wide raised beds with 120.0 cm spacing between beds center-to-center. Drip irrigation lines were installed before covering the beds with black plastic mulch. Hybrids were grown in four-plant plots arranged in a randomized complete blocks experiment design with three complete blocks (four-plant plot replications) per entry. The bare-root plants (clones) of these entries (5,988 clones per year) were produced in a low-elevation nursery (Winters, CA). Clones were harvested in Winters and transplanted in Salinas in early November through circular planting holes spaced 30.0 cm apart within and between rows (equivalent to a density of 45,000 plants/hectare). Experimental units were randomized each year. These experiments covered approximately 0.3 ha/year. They were irrigated, fertilized, sprayed with pesticides as needed, and managed according to the production practices used by Garcia Farms at Spence Ranch, Salinas, CA.

### Phenotyping

Ripe fruit were harvested from each plot once per week for 11–13 successive weeks from the beginning of April to the end of June in the 2017–2018 and 2018–2019 seasons, respectively. The harvest period was chosen to avoid the confounding effects of photoperiod sensitivity. The last harvest in both years was one week past the summer solstice (June 21), when daylengths reached their maximum (14.7 hours) and short-day hybrids typically ceased flowering at that latitude (Salinas, CA). The short-day hybrids in our experiment produced fruit through the summer solstice from flowers produced approximately four weeks earlier. Fruit yield (g/plant), count (number of fruit/plant), and weight (g/fruit) were recorded at each harvest (123,012 phenotypic observations were collected and analyzed for these traits and are available in Supplementary Files 3–6).

We sampled three fruit/plant from the sixth and twelfth harvest each year for fruit quality trait phenotyping using previously described methods (Petrasch *et al*. 2022). Fresh fruit were phenotyped on the day of sampling. The firmness of freshly harvested ripe fruit (maximum resistance kg-force) was assessed using a TA.XT plus Texture Analyzer with a TA-53 3 mm puncture probe (Stable Micro Systems Ltd., Goldaming, United Kingdom). Freshly harvested ripe fruit samples were frozen at $-20^{\circ }$C in Whirl-Pak Homogenizer Blender Filter Bags (Nasco, Fort Atkinson, WI, USA). Thawed and homogenized juice was sampled for measuring titratable acidity (TA; %), total soluble solid content (TSS; We sampled three fruit/plant from the sixth and twelfth harvest ^∘^BRIX), and total anthocyanin concentration (ANC; *μ*g/mL). TA percentages were measured with a Metrohm Robotic Titrosampler System from 1 to 5 mL of the defrosted homogenized fruit juice (Metrohm AG, Herisau, Switzerland). TSS was measured from approximately 200 *μ*L of juice on an RX-5000*α*-Bev Refractometer (ATAGO Co. Ltd., Tokyo, Japan). Total ANC was measured from a 25 *μ*L sample of juice in 200*μ*L 1% HCl in methanol by reading absorption at a wavelength of 520 nm on a Synergy HTX plate reader equipped with Gen5 software (Molecular Devices, San Jose, California, USA). A standard curve (y=sx+i) was calculated for quantifying ANC using a dilution series of pelargonidin (Sigma Aldrich, St. Louis, MI, USA) from zero to 300 *μ*g/mL in 50 *μ*g/mL increments, where *y* were absorption readings for the pelargonidin dilution series, *s* was the slope, *x* was the concentration of pelargonidin in the dilution series, and *i* was the intercept. ANC was estimated by (A−i)/s, where *A* was the absorption reading. We collected and analyzed 25,630 phenotypic observations for TSS, TA, ANC, and firmness (Supplementary Files 3–6).

### SNP genotyping

Single nucleotide polymorphisms (SNPs) for the genome-wide association and heterosis studies reported here were physically anchored to the chromosome-scale haplotype-phased assembly of the genome for the cultivar “Royal Royce” (https://phytozome-next.jgi.doe.gov/info/FxananassaRoyalRoyce_v1_0) and genotyped using a 50K Axiom array (Hardigan *et al*. 2020). The “Royal Royce” reference genome (https://phytozome-next.jgi.doe.gov/info/FxananassaRoyalRoyce_v1_0) was annotated using the chromosome nomenclature proposed by Hardigan *et al*. (2020). That chromosome nomenclature has been cross-referenced to previously published linkage group and chromosome nomenclatures (Hardigan *et al*. 2020; see Supplementary File 7).

DNA was isolated from the parents and hybrids using previously described protocols (Feldmann *et al*. 2024). Newly emerged leaves were harvested from field-grown seedlings at WEO in January 2017 for DNA isolation. Leaf tissue was placed into 1.1 mL tubes, freeze-dried in a Benchtop Pro (VirTis SP Scientific, Stone Bridge, NY), and ground using stainless steel beads in a Mini 1600 (SPEX Sample Prep, Metuchen, NJ). Genomic DNA (gDNA) was extracted from powdered leaf samples using the E-Z 96 Plant DNA Kit (Omega Bio-Tek, Norcross, GA, USA) according to the manufacturer’s instructions. To enhance the quality of the DNA and reduce polysaccharide carry-through, the protocol was modified with a Proteinase K treatment, a separate RNase treatment, an additional spin, and heated incubation steps during elution. DNA quantification was performed using Quantiflor dye (Promega, Madison, WI) on a Synergy HTX (Biotek, Winooski, VT).

The parent and hybrid DNA samples were genotyped with a 50 K Axiom SNP Array (Hardigan *et al*. 2020). SNP genotyping was performed by Affymetrix (Santa Clara, CA) on a GeneTitanTM HT Microarray System using DNA samples that passed quality and quantity control standards. SNP genotypes were automatically called with the Affymetrix Axiom Analysis Suite software (v1.1.1.66, Affymetrix, Santa Clara, CA). Samples with a call-rate greater than 90% were retained. The quality metrics output by the Affymetrix Axiom Analysis Suite and custom R scripts were utilized to filter out SNPs with MAF<0.05% and >10% missing data, which resulted in a SNP genotype file with 28,523 high-quality SNPs. We used the “*popgen()*” function from the “*snpReady*” R package (Granato and Fritsche-Neto 2018) to estimate observed heterozygosity, given as:


$$H_{o,i}=n_{AB,i}/n_{marker,i}$$

where $n_{AB}$ is the number of nonmissing heterozygous genotypes for the *i*th individual and the coefficient of inbreeding estimated as excess of homozygous relative to the expected for each genotyped hybrid is:


$$F_{i}=\frac{H_{o,i}-E(H)}{m-E(H)}$$

where $H_{o,i}$ is the observed heterozygosity in the target individual, E(H) is the expected heterozygosity in the population, and *m* is number of SNP markers (Weir and Cockerham 1984; Keller *et al*. 2011).

### Statistical analyses

The genomic relationship matrix (GRM; $K_{A}$) was estimated for parents and hybrids using the “*Amat()*” function in “*rrBLUP*” (Endelman 2011) from the 28,523 filtered SNPs. The dominance relationship matrix ($K_{D}$) was calculated with the “*sommer::D.mat()*” function in the “*sommer*” R package. We applied principal component analysis (PCA) to the SNP matrix using the built-in R function “*prcomp()*”. Scores from the first two principal components were extracted and plotted for every individual using the R package “*ggplot2*” (Wickham 2016).

Estimated marginal means (EMMs) for parents and hybrids were estimated for each trait within and between years using the R package “*emmeans*” (Lenth 2021). Linear mixed models (LMMs) were constructed and analyzed using the R package “*lme4*” (Bates *et al*. 2015), where hybrid (H) was analyzed as a fixed effect and block (B), year (Y), hybrid × year (H × Y), and the residual were analyzed as random effects in the across year analysis. Variance components for random effects were estimated using REML with the R package “*lme4*” (Bates *et al*. 2015). Mid-parent heterosis (MPH) was estimated by $\left({\bar{y}}_{F1}-{\bar{y}}_{MP}\right)$, where ${\bar{y}}_{MP}=({\bar{y}}_{P1}+{\bar{y}}_{P2})/2$, ${\bar{y}}_{P1}$ is the EMM for one parent, ${\bar{y}}_{P2}$ is the EMM for the other parent, and ${\bar{y}}_{F1}$ is the EMM for the hybrid between the two parents. Percent MPH was estimated by $({\bar{y}}_{F1}-{\bar{y}}_{MP})/{\bar{y}}_{MP}\times 100$. MPH was only estimated if EMMs were available for each of the two parents. Best-parent heterosis (BPH) was estimated by $\left({\bar{y}}_{F1}-{\bar{y}}_{BP}\right)$, where ${\bar{y}}_{BP}$ is the EMM for the best-parent. Percent BPH was estimated by $({\bar{y}}_{F1}-{\bar{y}}_{BP})/{\bar{y}}_{BP}\times 100$. For hybrids between two elite parents, the parent with the greater mean was identified as best. For hybrids between elite and exotic parents, the elite parent was identified as best. Contrasts between hybrid and best-parent EMMs and hybrid and mid-parent EMMs were estimated for every parent-hybrid combination using the R package “*emmeans*” (Lenth 2021).

LMM analyses were repeated with hybrid analyzed as a random effect to estimate the among-hybrid variance component and broad-sense heritability on a clone-mean basis


(1) $${\hat{H}}^{2}={\hat{\sigma }}_{G}^{2}/{\hat{\sigma }}_{\bar{P}}^{2}$$

where ${\hat{\sigma }}_{G}^{2}$ is the among hybrid variance


(2) $${\hat{\sigma }}_{\bar{P}}^{2}={\hat{\sigma }}_{G}^{2}+{\hat{\sigma }}_{G\times Y}^{2}/y+{\hat{\sigma }}_{E}^{2}/ry$$

is the phenotypic variance on a clone-mean basis, ${\hat{\sigma }}_{G\times Y}^{2}$ is the hybrid × year variance, ${\hat{\sigma }}_{E}^{2}$ is the residual variance, *y* is the number of years, and *r* is the harmonic mean number of replications. The among hybrid variance estimated from clones has the same expected causal genetic variances as identical twins, specifically 100% of the additive and nonadditive genetic variances (Lynch and Walsh 1998).

We regressed EMM, BPH, and MPH on the genome-wide estimate of heterozygosity (H) using the “lm()” function from the R package “*stats*” (R Core Team 2023), with both linear and quadratic terms, and only the linear term to estimate the trait-heterozygosity correlation (David 1998; Hansson and Westerberg 2002; Grueber *et al*. 2008; Chapman *et al*. 2009; Szulkin *et al*. 2010). We used the “anova()” function to compare the nested linear model to determine if the 2-degree model fit significantly better than the 1-degree model. This analysis was conducted in the full population, and in the elite×elite population to compare the effect of the elite × exotic hybrids on these interpretations.

The R package “*sommer*” (Covarrubias-Pazaran 2016) was used to estimate the additive genetic covariance between pairs of mean-centered traits (EMMs) and scaled to unit variance. The correlation is equal to the covariance of two mean-center and unit-scaled variables. The Z-ratio reported by “*sommer*” was used to estimate the *P*-value using “*pnorm(Zratio, lower.tail=F)*” in R.

We used “*sommer*” (Covarrubias-Pazaran 2016) to estimate directional dominance using individual homozygosity ($H_{o}$) as a covariate in LMM analyses (Xiang *et al*. 2016; Endelman 2023). For each trait, we fit the models


(3) $$Y=\mu +H_{o}+A+D+\epsilon$$

and


(4) $$Y=\mu +H_{o}+H_{o}^{2}+A+D+\epsilon ,$$

where *Y* is the trait, BPH, or MPH of a hybrid, $H_{o}$ is the observed array heterozygosity, *A* are the additive genetic values and $A∼N(0,K_{A}\sigma_{A}^{2})$, *D* are the dominance genetic values and $D∼N(0,K_{D}\sigma_{D}^{2})$, and ϵ are the residuals and $\epsilon ∼N(0,I\sigma_{\epsilon }^{2})$. We used the “anova()” function to compare the two linear models to determine if the 2-degree model fit significantly better than the 1-degree model for the covariate for individual homozygosity ($H_{o}$). This analysis was performed in support of the trait-heterozygosity correlation analysis.

The effective population size ($N_{e}$) of genotyped accessions was estimated from the change in heterozygosity over generations (Crow and Kimura 1970; Frankham *et al*. 2002):


(5) $$N_{e}=\frac{-1}{2\cdot \ln\;\left(\frac{H_{1}}{H_{0}}\right)}$$

where *H* is the estimated heterozygosity obtained from SNP genotyping data at between the parents ($H_{0}$) and the progeny ($H_{1}$). We performed this calculation twice, once using all parents and all progeny ($N_{e,All}$) and again using only those parents of strictly University of California, Davis (UCD) descent and the progeny in families where both parents are of strictly UCD descent ($N_{e,elite}$).

We applied DNA forensic approaches for diploid organisms to the problem of identifying parents and authenticating pedigrees in allo-octoploid strawberry, as in Pincot *et al*. (2021). Genotypic transgression ratios were estimated for all possible duos and trios of individuals in two study populations (described above) from genotypes of multiple SNP marker loci. For parent–offspring (PO) duos of individuals in the SNP profile database for a population, the genotypic transgression score for the *i*th SNP marker was estimated by


(6) $$S_{i}=f(AA_{O_{i}})\cdot f(BB_{P_{i}})+f(BB_{O_{i}})\cdot f(AA_{P_{i}})$$

where i=1,2,…,m, m=number of SNP marker loci genotyped in each pair of probative DNA samples, $f(-{-}_{O_{i}})$ is the frequency of a homozygous genotype (coded *AA* and *BB*) in the candidate offspring individual and $f(-{-}_{P_{i}})$ is the frequency of a homozygous genotype in the candidate parent individual (similarly coded *AA* and *BB*) for the *i*th SNP marker locus. This equation was applied to a single pair of candidate individuals at a time and was thus constrained to equal 0 or 1; hence, $S_{i}=0$ when homozygous genotypes were identical for a pair of individuals and $S_{i}=1$ when homozygous genotypes were different for a pair of individuals. Duo transgression ratios (DTRs) were estimated for every pair of individuals in the population by summing $S_{i}$ estimates from equation (6) over *m* marker loci:


(7) $$\mathrm{DTR}=\frac{1}{m}\sum_{i=1}^{m}S_{i}$$

For trios of individuals in the SNP profile database for a population, the genotypic transgression score for the *i*th SNP marker was estimated by


(8) $$\begin{array}{rl} T_{i} & =f(AB_{O_{i}})\cdot f(AA_{P1_{i}})\cdot f(AA_{P2_{i}})\\ & \;+f(AB_{O_{i}})\cdot f(BB_{P1_{i}})\cdot f(BB_{P2_{i}}) \end{array}$$

where $f(AB_{O_{i}})$ is the frequency of a heterozygous genotype (coded *AB*) in the candidate offspring individual, $f(-{-}_{P1_{i}})$ is the frequency of either homozygous genotype (*AA* or *BB*) in candidate parent 1 (P1), and $f(-{-}_{P2_{i}})$ is the frequency of either homozygous genotype in candidate parent 2 (P2) for the *i*th SNP marker locus. Trio transgression ratios (TTRs) were estimated for every parent–parent–offspring (PPO) trio of individuals in the population by summing $T_{i}$ estimates from equation (8) over *m* marker loci:


(9) $$\mathrm{TTR}=\frac{1}{m}\sum_{i=1}^{m}T_{i}+S1_{i}+S2_{i}-S1_{i}\cdot S2_{i}$$

where *m* is the number of SNP marker loci genotyped for a trio of individuals, $S1_{i}$ is the score estimated from equation (6) for candidate parent 1, and $S2_{i}$ is the score estimated from equation (6) for candidate parent 2. To avoid double counting transgressions, TTR estimates were corrected by subtracting $S1_{i}$ × $S2_{i}$.

## Results and discussion

### Genetically distinct groups are lacking within the California population

**Fig. 1.**
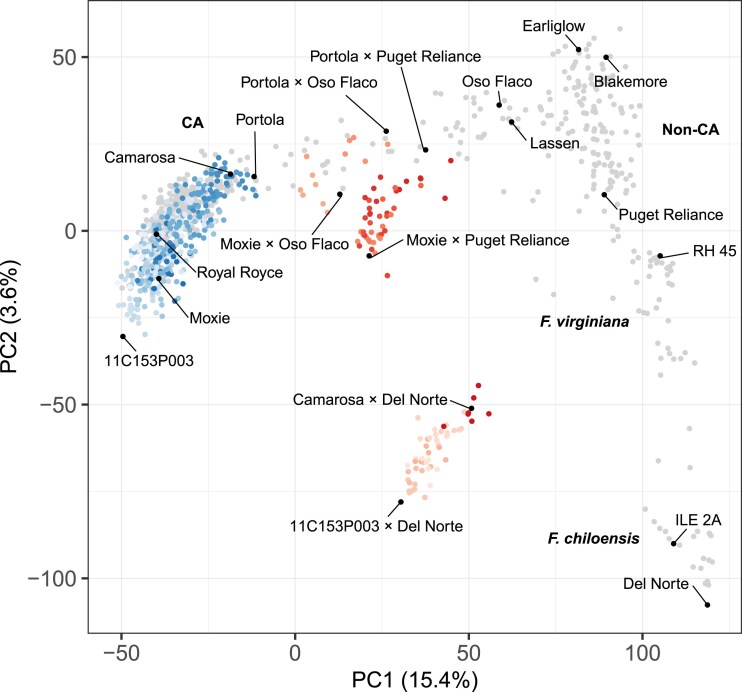
Genetic diversity of elite and exotic parents and elite×elite and elite×exotic hybrids. Genetic relationships were estimated among 27 elite parents, three exotic parents, 356 elite×elite hybrids, 113 elite×exotic hybrids, and 581 additional F.×ananassa, *F. virginiana*, and *F. chiloensis* individuals from 28,513 SNPs genotyped with a 50K Axiom array. The scatterplot displays the first two scores (PC1 and PC2) from a principal component analysis of the genetic relationship matrix for elite (CA) parents and elite×elite (CA×CA) hybrids (blue circles), elite×exotic (CA × exotic) hybrids (red circles), and 385 other CA and 196 non-CA population individuals (gray circles). The different shades of blue and red depict different full-sib families.

The domestication of strawberry for large-scale production over the last 70 years has been driven by direct selection for increased fruit yield, weight, and firmness among hybrid individuals, predominantly within closed populations, as exemplified by the California population, a progressively improved series of cultivars and other asexually propagated hybrid individuals developed at the University of California, Davis since the 1950s. Those cultivars played a critical role in the strawberry Green Revolution in California where genetic gains for yield are estimated to have surpassed 2,800% (Feldmann *et al*. 2024). The parents of the elite×elite hybrids developed for the present study were selected to survey genetic variation within the California population, which constituted a collection of 1,734 hybrid individuals preserved by asexual propagation when our studies were undertaken in 2015 (Fig. 1; Supplementary File 1). Their selection was informed by previous breeding history and genetic diversity analyses (Hardigan *et al*. 2021; Pincot *et al*. 2021). We knew from forensic reconstruction of pedigree records and genetic diversity analyses that the parents and other individuals preserved in the 2015 rendition of the California population were hybrids that originated between 1924 and 2011 (Hardigan *et al*. 2021; Pincot *et al*. 2021), but little else because their phenotypic characteristics were previously undocumented, a deficiency rectified by the present study and several companion studies (Pincot *et al*. 2018, 2020, 2022; Jiménez *et al*. 2022, 2024; Petrasch *et al*. 2022; Feldmann *et al*. 2023, 2024; Knapp *et al*. 2023).

Figure 1 illustrates the genetic diversity of the 27 elite (California population) parents and 356 hybrids among them (Supplementary Fig. 1). To compare the genetic diversity of those parents and hybrids to global genetic diversity (Hardigan *et al*. 2020, 2021; Feldmann *et al*. 2024), 385 additional CA population individuals (developed between 1927 and 2012) and 196 North American and European cultivars and ecotypes of *F. virginiana* and *F. chiloensis* were included in our principal component analysis of the genetic relationship or G matrix (Fig. 1; Supplementary File 1). The G matrix was estimated using 28,513 array-genotyped SNPs (Hardigan *et al*. 2020).

The oldest elite parent was “Camarosa” (developed in 1988), an important cultivar at the foundation of the modern (post-1970) CA population (Fig. 1). Our principal component analysis (PCA) corroborated that the elite parents and hybrids and other post-1970 California population individuals constitute a single genetically contiguous group (blue points in Fig. 1 identified as the CA group). They formed an elliptically shaped cluster of individuals bounded by “Camarosa” at the upper vertex and 11C153P003 at the lower vertex (Fig. 1). Using photoperiod sensitivity phenotypes observed in field studies and array-genotyped SNPs in linkage disequilibrium with the *PERPETUAL FLOWERING* locus (Feldmann *et al*. 2024), we found that photoperiod sensitive (short-day flowering) and photoperiod insensitive (day-neutral flowering) individuals are highly admixed and randomly distributed within the CA group (Supplementary Fig. 1). There is no evidence that separate photoperiod sensitivity groups ever emerged or existed in the California population.

As previously reported (Hardigan *et al*. 2021; Pincot *et al*. 2021), PC1 × PC2 coordinates of individuals within the CA group are positively correlated with their chronological ages, with younger individuals near the lower vertex (e.g. 11C153P003) and older individuals near the upper vertex (e.g. “Camarosa”) (Fig. 1; Supplementary Fig. 1). “Camarosa” and “Portola” identify the boundary between modern CA population individuals and chronologically older CA population individuals, including founders dispersed horizontally to the right with “Oso Flaco” and “Lassen” farthest from the boundary (Fig. 1). “Oso Flaco” and “Lassen” are proximal to an admixed group of North American and European cultivars bounded by “Earliglow”, “Blakemore”, and “Puget Reliance” that we previously described as a “cosmopolitan” group (Hardigan *et al*. 2021). The octoploid wild relatives of cultivated strawberry formed two well separated groups distal to the cosmopolitan group, starting with *F. virginiana* ecotypes anchored by RH 45 at the upper vertex closest to the cosmopolitan group and finishing with *F. chiloensis* ecotypes anchored by Del Norte at the lower vertex farthest from the cosmopolitan group (Fig. 1).

### Creating *ad hoc* heterotic groups and patterns to uncover and restore heterosis in strawberry

Using insights gained from previous analyses of breeding history and genetic diversity (Gil-Ariza *et al*. 2009; Horvath *et al*. 2011; Sánchez-Sevilla *et al*. 2015; Vallarino *et al*. 2018; Hardigan *et al*. 2021; Pincot *et al*. 2021), we knew that *ad hoc* heterotic patterns had to be created using genetically divergent parents to study and uncover heterosis in cultivated strawberry (Melchinger and Gumber 1998). Three exotic parents (“Oco Flaco”, “Puget Reliance”, and “Del Norte”) were selected to develop elite×exotic hybrids for the *ad hoc* heterotic patterns explored in our study: elite×elite (n=356), elite × “Puget Reliance” (n=47), elite × “Oso Flaco” (n=14), and elite × “Del Norte” (n=52) (Fig. 1; Supplementary File 1). We identified elite×elite as a heterotic pattern, even though our principal component analysis substantiated the absence of distinct groups of individuals within the California population (Fig. 1; Supplementary Fig. 1). Hybrids from the elite×elite heterotic pattern were the benchmark by which hybrids from elite×exotic heterotic patterns were compared.

Figure 1 illustrates the patterns of genetic diversity uncovered by our principal component analysis of genetic relationships among elite and exotic parents and their hybrids (Supplementary File 1). The three exotic parents populate the right arm of the inverted V-shaped principle component distribution spanned by “Oso Flaco” at the upper and “Del Norte” at the distal ends of the arm (Fig. 1). “Oso Flaco” and “Lassen” demarcate the border between early California and non-California population individuals. Three distinct groups of elite×exotic hybrids (heterotic patterns) were observed with coordinates halfway between their respective heterotic groups, e.g. hybrids for the elite × “Del Norte” heterotic pattern were located midway between the California and *F. chiloensis* heterotic groups (Fig. 1). Genetic distances between the elite×elite hybrids (blue points in Fig. 1) and elite × exotic hybrids (red points in Fig. 1) increased as the exoticness of the exotic parent increased. Genetic distances within and between groups were strongly correlated with the positions of the 27 elite and three exotic parents in the global genetic diversity analysis (Fig. 1).

The exotic parents we selected were known to be genetically distant to individuals within the California population and were deliberately selected to sample progressively greater doses of exotic alleles across the domestication spectrum, from an early California population founder (“Oso Flaco”) to a non-California population cultivar (“Puget Reliance”) to “Del Norte” (PI551753), an *F. chiloensis* subsp. *lucida* ecotype native to coastal Washington (Fig. 1; Supplementary File 1). “Puget Reliance” (PI664321) is a Pacific Northwest-adapted, short-day cultivar developed in 1994. “Oso Flaco” (55C023P001) is a backcross-derived descendant of a hybrid between the recurrent parent “Lassen” (developed in 1936) and an extinct *F. chiloensis* subsp. *lucida* ecotype native to the Guadalupe-Nipomo Dunes near Oso Flaco Lake, Guadalupe, California. “Oso Flaco” and “Lassen” are proximal to one another in the group of early California population founders, whereas “Puget Reliance” resides further downstream in the admixed, cosmopolitan group of North American and European cultivars (Fig. 1; Hardigan *et al*. 2021; Pincot *et al*. 2021).

### The genomes of high yielding California population hybrids are moderately to highly inbred

**Fig. 2.**
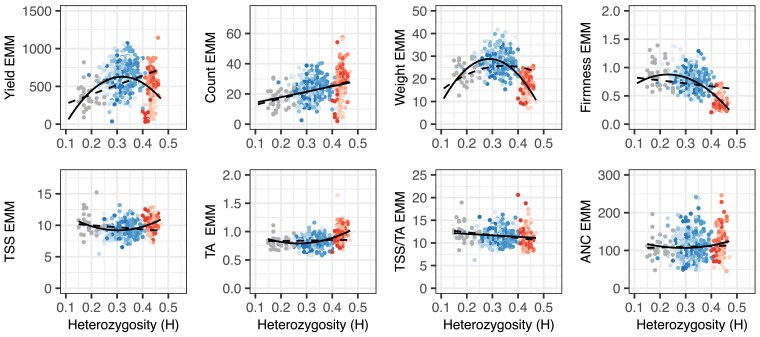
Heterozygosity (H) by phenotypic mean distributions for fruit yield, count, weight, firmness, TSS, TA, TSS/TA, and ANC among 31 ${\text{S}}_{1}$ offspring of nine elite parents (gray points), 356 elite×elite hybrids (blue points), and 113 elite×exotic hybrids (red points) grown in Salinas, California field experiments in 2017–2018 and 2018–2019. The EMMs of hybrids and S_1_ offspring were estimated from phenotypes observed across harvests, replicates, and years. Heterozygosity was estimated from a genome-wide sample of 28,513 SNPs genotyped with a 50K Axiom array. The shades of red and blue depict hybrids within different full-sib families. The solid black lines are predicted values from linear or quadratic regressions of EMMs on H. The dashed black lines are predicted values from linear or quadratic regressions of EMMs on H using a linear model with additive and dominance genetic relationships incorporated.

Our analyses show that modern California population hybrids, including “Royal Royce” and several other high yielding cultivars (asexually propagated hybrid individuals), are moderately to highly inbred (Fig. 2; Supplementary File 1). Using a genome-wide sample of 50K array-gentoyped SNPs, heterozygosity (H) was estimated to range from 0.30 to 0.41 among the elite parents and from 0.21 to 0.41 among the elite×elite hybrids (blue points in Figs. 1 and 2; Supplementary File 1). None of the elite×elite hybrids were more heterozygous than the most heterozygous elite parent (“Portola”; H=0.41); however, 21.1% were more inbred (*H* ranged from 0.21 to 0.29 with a mean of 0.27) than the most inbred elite parent (11C180P001; H=0.30). These estimates show that the genomes of modern California population hybrids are 59–79% inbred and that “effective selfing rates” have increased as individuals within the California population have become increasingly more inbred (Ritland 1984; Hardigan *et al*. 2021). The absence of elite×elite hybrids with increased heterozygosity and presence of elite×elite hybrids with decreased heterozygosity suggests that the inbred fractions of the elite parent genomes are mostly shared in common (Fig. 2).

The inclusion of only three exotic parents doubled effective population size ($N_{e}$), which was estimated for hybrids developed with and without exotic parents using the observed change in heterozygosity (*H*) between the parent (t=0) and hybrid (t=1) generations (Crow and Kimura 1970). The $N_{e}$ estimate for the elite parents was ($N_{e}=3.91$; $H_{t=0}=0.355$; $H_{t=1}=0.313$) was 9-fold smaller than the census number ($N_{c}=27$). $N_{e}$ increased to 7.95 when the three exotic parents were added ($H_{t=0}=0.357$; $H_{t=1}=0.335$), a near linear increase proportional to $N_{c}$. Small $N_{e}$ estimates like these seemingly contradict our proposal that domesticated strawberry populations harbor massive allelic diversity. Can both be true? Can the small $N_{e}$-genetic diversity paradox be reconciled?

### Despite progressive inbreeding, present-day California population hybrids are heterozygous for the equivalent of a typical diploid genome or more

We assembled haplotype-phased reference genomes for a high yielding modern cultivar (“Royal Royce”) to shed light on the small $N_{e}$-genetic diversity paradox and inbreeding in a high yielding modern cultivar (https://phytozome-next.jgi.doe.gov/info/FxananassaRoyalRoyce_v1_0). On the one hand, founder effects and the small $N_{e}$ predict a significant reduction of genetic variation through the random fixation and loss of alleles and consanguineous matings alone (Crow and Kimura 1970; Nei and Tajima 1981; Barton and Charlesworth 1984; Sjulin and Dale 1987; Dale and Sjulin 1990; Pincot *et al*. 2021). On the other hand, genome-wide analyses have uncovered massive nucleotide diversity in the noninbred factions of the genomes of F.×ananassa individuals (Hardigan *et al*. 2021). Despite the inbreeding that has accumulated within the California population since the 1950s (Figs. 1 and 2; Hardigan *et al*. 2021; Pincot *et al*. 2021), our analyses suggest that the noninbred fractions of “Royal Royce” and other modern California population hybrids harbor thousands of segregating genes and millions of segregating DNA variants transmitted by a comparatively small number of founders. We suspect that much of this genetic variation persists in the form of unfavorable, incompletely dominant alleles that diminish hybrid performance and have not yet been purged by breeding, even in the most highly domesticated populations (Gore *et al*. 2009; Yang *et al*. 2017; Hardigan *et al*. 2021; Lozano *et al*. 2021; Feldmann *et al*. 2024).

The study described here was underway before the octoploid strawberry genome was sequenced and assembled (Edger *et al*. 2019). That assembly was developed for “Camarosa”, an important California population cultivar (hybrid individual) and one of the 27 elite parents used in our study (Supplementary File 1). The “Camarosa” assembly was unphased and imperfect, but provided the highly contiguous physical genetic framework needed to estimate the genome-wide diversity of the hybrid species (Edger *et al*. 2019; Hardigan *et al*. 2021). Hardigan *et al*. (2020) used the “Camarosa” genome assembly to design the 50 K SNP array used to genotype the parents and hybrids in our study (Supplementary File 1). With that resource in place, we discovered that “Camarosa” was the second-most heterozygous elite parent (H=0.40) among the 27 elite parents (*H* ranged from 0.30 to 0.41; Supplementary File 1). The most heterozygous elite parent was “Portola” (H=0.41). As shown later, both turned out to be critical for discovering remnants of heterosis for fruit yield and yield components within the California population.

Within two years of the octoploid genome assembly breakthrough (Edger *et al*. 2019), PACBIO introduced the highly accurate long-read HiFi sequencing technology that revolutionized the phasing and assembly of complex genomes (Hon *et al*. 2020; Michael and VanBuren 2020; Gladman *et al*. 2023). That technology was initially tested on octoploid strawberry and four other species with complex genomes (Hon *et al*. 2020). As part of that collaboration, we developed the earliest haplotype-phased assemblies of the octoploid genome of strawberry using trio binning (Koren *et al*. 2018) and DNA of the cultivar “Royal Royce” (FaRR1; https://phytozome-next.jgi.doe.gov/info/FxananassaRoyalRoyce_v1_0), one of the elite parents used in our study and the highest yielding cultivar identified in a study of historical genetic gains in the California population (Feldmann *et al*. 2024). Assemblies for both haplotyes were publicly released in 2020 during the COVID pandemic (https://phytozome-next.jgi.doe.gov/info/FxananassaRoyalRoyce_v1_0, and were used in the present study to estimate the inbreeding coefficient for “Royal Royce” and the gene content found in the noninbred fractions of the genomes of “Royal Royce” and other CA population hybrids.

Our estimate of the inbreeding coefficient for the cultivar “Royal Royce” from array-genotyped SNPs (F=1−H=0.62) was slightly lower than the fraction of the genome (0.69) estimated to be identical-by-descent (IBD) from trio binning long-read DNA sequences (Supplementary File 1; https://phytozome-next.jgi.doe.gov/info/FxananassaRoyalRoyce_v1_0; Koren *et al*. 2018; Hon *et al*. 2020). Using the estimated F range for elite×elite hybrids (0.59–0.79), “Royal Royce” and other highly yielding CA population hybrids were estimated to be heterozygous for 20,370–44,280 of the 97,000–108,000 genes identified by different octoploid genome annotations (Edger *et al*. 2019; Jin *et al*. 2023; https://phytozome-next.jgi.doe.gov/info/FxananassaRoyalRoyce_v1_0). The lower end of that range is slightly lower than number of genes found in most diploid plants (Lynch 2007; Michael and Jackson 2013; Kersey 2019); hence, despite being 59–79% inbred, “Royal Royce” and the other California population hybrids we studied are heterozygous (segregating) for the number of genes found in a typical diploid genome or more (Lynch 2007; Michael and Jackson 2013; Kersey 2019). To put this into perspective, these estimates are equivalent to a hybrid between two inbred lines being heterozygous for every gene in a diploid organism, assuming a random distribution of DNA variants. The genetic variation that has persisted in modern cultivars, despite founder effects and the small $N_{e}$, is one of the more compelling insights about strawberry domestication that has emerged since the octoploid genome was first sequenced and nucleotide diversity could be surveyed genome-wide (Edger *et al*. 2019; Hardigan *et al*. 2021).

### Heavy genetic loads persist for heterotic traits within the California population

The residual heterozygosity uncovered by our analyses suggested that heavy genetic loads persisted for heterotic traits in the noninbred fractions of the genomes of California population individuals (Table 1). We tested this by phenotyping 31 self-pollinated (${\text{S}}_{1}$) offspring of nine of the 27 elite (${\text{S}}_{0}$) parents (Table 1; gray points in Fig. 2). Using array-genotyped SNPs, heterozygosity (H) was estimated to decrease 47.1% among ${\text{S}}_{1}$ individuals, close to the theoretical expectation of 50.0% (H ranged from 0.15 to 0.25 with a median of $\tilde{y}=0.18$ among ${\text{S}}_{1}$ individuals; Supplementary File 1). Statistically significant inbreeding depression (${\bar{y}}_{S_{0}}>{\bar{y}}_{S_{1}}$) was observed for fruit yield, count, and weight, the three traits predicted to be heterotic and therefore most likely depressed by inbreeding, where ${\bar{y}}_{S_{0}}$ is the estimated marginal mean (EMM) among S_0_ parents and ${\bar{y}}_{S_{1}}$ is the EMM among S_1_ offspring (Table 1). Fruit yield, the trait most severely depressed by inbreeding, decreased 51.6% among S_1_ individuals. We attributed the inbreeding depression observed for these traits to the effects of incompletely dominant unfavorable (deleterious) alleles masked by heterozygosity in the parents, and concluded that CA population individuals carry heavy loads of deleterious alleles for fruit yield, count, and weight in the noninbred fractions of their genomes (Lynch 1991; Charlesworth and Willis 2009; Mackay *et al*. 2021).

**Table 1.** The effect of inbreeding on fruit yield and quality traits within the California population.

Trait	${\bar{y}}_{S_{0}}$	${\bar{y}}_{S_{1}}$	Contrast	Pr>F	Change (%)
Heterozygosity (H)	0.356	0.192	0.164	<0.0001	− 46.1
Yield (g/plant)	727.6	369.6	358.0	<0.0001	− 49.2
Count (fruit)	24.5	16.4	8.2	<0.0001	− 33.1
Weight (g/fruit)	29.7	22.8	6.9	<0.0001	− 23.2
Firmness (kg-Force)	0.81	0.86	− 0.05	0.32	6.2
TSS (%)	8.9	10.2	− 1.3	<0.0001	14.6
TA (%)	0.75	0.81	− 0.06	0.01	8.0
TSS/TA	11.9	12.7	− 0.8	0.08	6.7
ANC (*μ*g/mL)	104.2	111.5	− 7.3	0.29	7.0

Contrasts (${\bar{y}}_{S_{0}}-{\bar{y}}_{S_{1}}$) were estimated between the phenotypic means of nine elite parents (S_0_ individuals) and 31 of their self-pollinated (S_1_) offspring for fruit yield, count, weight, firmness, TSS, TA, and ANC, where ${\bar{y}}_{S_{0}}$ is the EMM of the nine parents and ${\bar{y}}_{S_{1}}$ is the EMM of the 31 S_1_ offspring. EMMs were estimated from phenotypes observed across clonal replicates, harvests, and years in Salinas, California field experiments in 2017–2018 and 2018–2019. The percent change was estimated by $({\bar{y}}_{S_{0}}-{\bar{y}}_{S_{1}})/{\bar{y}}_{S_{0}}\times 100$.

The four fruit quality traits phenotyped in our study (firmness, TSS, TA, or ANC) were not depressed by inbreeding (Table 1; gray points in Fig. 2). The phenotypic means of the S_1_ offspring were actually greater than their ${\text{S}}_{0}$ parents for these traits; however, these differences between the ${\text{S}}_{0}$ and ${\text{S}}_{1}$ means were only statistically significant for TSS and TA (Table 1). These increases suggest that recessive or incompletely dominant favorable alleles masked by heterozygosity in the parents were exposed by inbreeding for these traits.

### The domestication of strawberry for large-scale production reshaped allelic variation nearly genome-wide

Figure 2 displays the heterozygosity and phenotypic variation of hybrids developed with and without exotic parents (red and blue points, respectively, in the H × phenotypic mean distributions). The heterozygosity range was greater for elite×exotic hybrids (0.40≤H≤0.47 with a median of $\tilde{y}=0.43$) than elite×elite hybrids (0.21≤H≤0.41 with a median of $\tilde{y}=0.32$) with minimal overlap between them. The least inbred elite parent (“Portola”; H=0.41) and least inbred elite×elite hybrid (10C122P003 × 01C206P005; H=0.41) were still more inbred than 99% of the elite×exotic hybrids screened in our study (Fig. 2; Supplementary File 1).

The domestication traits phenotyped in our study were quantitative and highly heritable. REML estimates of narrow-sense heritability ($h^{2}$) ranged from 0.57 to 0.68 for fruit yield, count, and weight and 0.75 to 0.81 for TA, TSS/TA, and ANC. The estimate for TSS was markedly lower ($h^{2}=0.32$). REML estimates of broad-sense heritability on a clone-mean basis ($H^{2}$) ranged from 0.53 to 0.94 for these traits. When across-year phenotypic means for elite S_1_ individuals and elite×elite and elite×exotic hybrids were regressed on heterozygosity (solid lines in Fig. 2), we observed positive parabolic trends for fruit yield, weight, and firmness, a positive linear trend for fruit count, and negative parabolic trends for TSS (or “sugars”), TA (or “acids”), and ANC. The shapes and slopes of these regression curves were consistent with additive genetic correlations among these traits, predictions from our genome-wide association studies (documented below), and insights gained from analyses of historical genetic gains in the California population where long-term direct selection significantly increased fruit yield, weight, and firmness and indirect selection significantly increased fruit count and decreased TSS and TA (Feldmann *et al*. 2024).

To assess the importance of directional dominance for the traits phenotyped in our study (Varona *et al*. 2018, 2022; Endelman 2023), the *H* × phenotypic mean regressions were repeated by fitting a genetic model with additive and dominance effects (the predicted values for those regressions are depicted by dashed lines in Fig. 2). We observed a positive linear slope for fruit yield and count, a positive and slightly parabolic slope for fruit weight, and a negative linear slope for fruit firmness (Fig. 2). The signs of those slopes were consistent with the presence or absence of inbreeding depression: fruit yield, count, and weight were depressed by inbreeding, whereas fruit firmness was not (Table 1). These results suggest that the fruit yield, count, and weight increases observed in the California population were caused by directional dominance of the underlying loci (Lamkey and Edwards 1999; Birchler *et al*. 2010; Schnable and Springer 2013; Endelman 2023). The slopes for fruit quality traits (TSS, TA, and ANC), none of which were depressed by inbreeding, were weakly negative and nearly flat (Table 1; Fig. 2), which suggested that directional dominance was less important or unimportant for these traits.

**Fig. 3.**
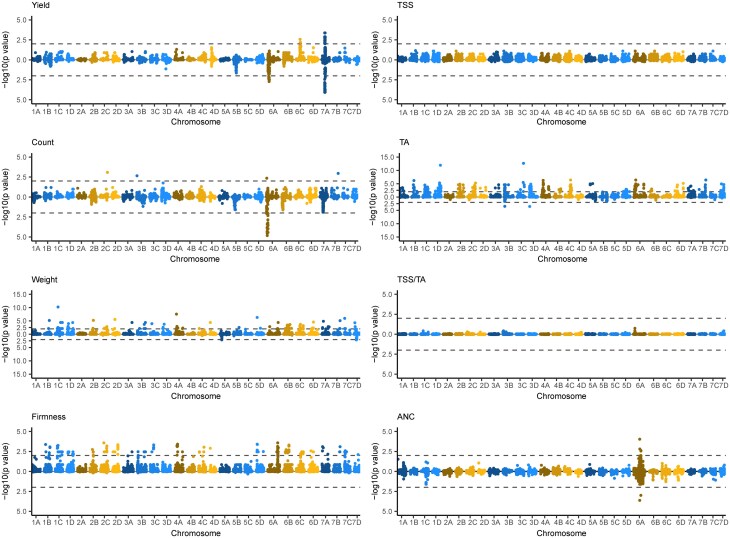
Genetic variants associated with fruit phenotypes identified by genome-wide association studies in strawberry. GWAS statistics were estimated for fruit yield, count, weight, firmness, TSS, TA, TSS/TA (sugar-to-acid ratio), and ANC from the EMM of the parents and hybrids observed over the 2017–2018 and 2018–2019 growing seasons in Salinas, CA. The upper Manhattan plot in each mirror image pair displays statistics for elite×elite and elite×exotic hybrids combined (n=469), whereas the lower Manhattan plot in each pair displays statistics for elite×elite hybrids only (n=356). The horizontal dashed lines depict genome-wide significance thresholds of $5\times 10^{-8}$.

Genome-wide association studies of hybrids developed with and without exotic parents showed that breeding has decreased genetic variation nearly genome-wide for traits under strong selection (Fig. 3; Supplementary File 8). This finding was predicted by genome-wide reductions of nucleotide diversity and heterozygosity within the California population (Hardigan *et al*. 2021). The phenotypic effects of the latter, however, were previously unknown. GWAS-estimated additive effects were greater for elite×exotic than elite×elite hybrids for every trait except ANC, the only trait that does not appear to have been under selection in the California population (Fig. 3; Supplementary File 8).

The Manhattan plots shown in the left-hand column of Fig. 3 illustrate the genome-wide effects of breeding on associations for traits that were directly selected in the CA population (fruit yield, weight, and firmness), in addition to fruit count, a trait that was indirectly selected and strongly positively genetically correlated with fruit yield. The additive genetic correlation between fruit yield and count was ${\hat{r}}_{G}=0.86$ among elite×elite hybrids. The Manhattan plots shown in the right-hand column of Fig. 3 illustrate the genome-wide effects of breeding on associations for fruit quality traits that were either indirectly selected (TSS and TA) or unselected (ANC) in the CA population. The addition of elite×exotic hybrids to the genome-wide association study (GWAS) population increased the additive effects of genetic variants nearly genome-wide for every trait except ANC, the only trait that does not appear to have been under direct or indirect selection in the CA population (Fig. 3; Supplementary File 8). The additive effect differences observed when hybrids with exotic parents were included or excluded were most pronounced and genome-wide for fruit weight, firmness, TSS, and TA (Fig. 3).

Statistically significant genetic variants were not observed for TSS; however, additive effects for TSS-associated genetic variants increased nearly genome-wide when elite×exotic hybrids were included in the analysis (Fig. 3; Supplementary File 8). Statistically significant genetic variants were discovered on 19 chromosomes for fruit weight, 23 chromosomes for fruit firmness, and 22 chromosomes for TA when elite×exotic hybrids were included in the analysis, and were virtually nonexistent when they were excluded from the analysis, apart from two small-effect loci for fruit weight on chromosomes 5A and 7D and two small-effect loci for TA on chromosomes 3B and 3D (Fig. 3; Supplementary File 8).

### Allelic variation for fruit firmness loci was restored genome-wide in hybrids between soft-fruited exotic and firm-fruited elite parents

The stark contrast in GWAS-estimated additive effects between elite×elite and elite×exotic hybrids was especially dramatic for fruit firmness (Fig. 3). We knew that a large-effect QTL associated with *POLYGALACTURONASE1* (*PG1*), a fruit softening gene found on chromosome 6A, was one of the loci targeted by selection for increased firmness in the California population (Jiménez *et al*. 2024). Jiménez *et al*. (2024) showed that a loss-of-function mutation in *PG1* more than doubled fruit firmness and that every UC cultivar developed since 1970 is homozygous for the favorable (mutant) *PG1* allele, including the UC cultivars used as parents in our study (Supplementary File 1). We observed a strong signal for that QTL among elite × exotic hybrids (Fig. 3). The SNPs most strongly associated with the QTL were AX-184023221, AX-184726882, AX-184210676, and AX-184242253. The latter two were among the most significant SNPs identified by Jiménez *et al*. (2024). Using those SNPs as proxies for *PG1* alleles, we discovered that the 27 elite parents were homozygous for the favorable *PG1* allele, whereas the exotic parents were either homozygous for the unfavorable *PG1* allele (“Del Norte” and “Puget Reliance”) or heterozygous (“Oso Flaco”). Hence, *PG1* did not segregate among elite×elite hybrids, but did among elite × exotic hybrids (Fig. 3). Although *PG1* was the only statistically significant locus identified for fruit firmness in previous genome-wide association studies Hardigan *et al*. (2021) and Jiménez *et al*. (2024), missing heritability, the abundance of statistically significant GWAS signals among elite×exotic hybrids in our study (Fig. 3; Supplementary File 2), and previous QTL mapping studies suggest that domestication has targeted multiple loci affecting fruit firmness (Cockerton *et al*. 2019; Lee *et al*. 2021; Munoz *et al*. 2024; Prohaska *et al*. 2024).

### A dominance hypothesis argument for historical genetic gains and the fixation of heterosis in the California population

**Fig. 4.**
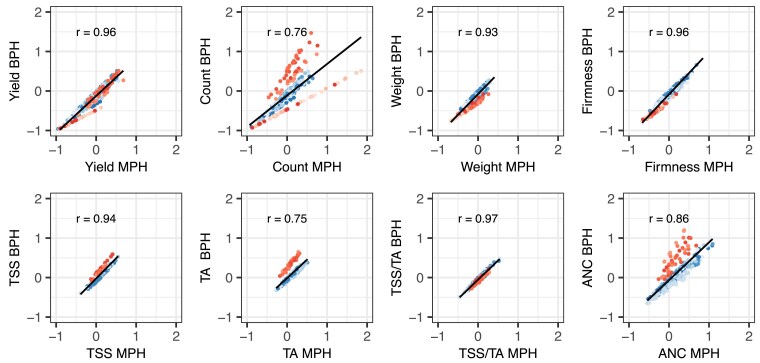
Correlations (r) between mid-parent heterosis (MPH) and best-parent heterosis (BPH) for fruit yield, count, weight, firmness, TSS, TA, TSS/TA, and ANC among strawberry hybrids (n=469) grown in Salinas, California field experiments in 2017–2018 and 2018–2019. H was estimated from a genome-wide sample of 28,513 SNPs genotyped with a 50K Axiom array. MPH ratios were estimated by $({\bar{y}}_{F1}-{\bar{y}}_{MP})/{\bar{y}}_{MP}$, where ${\bar{y}}_{MP}=({\bar{y}}_{P1}+{\bar{y}}_{P2})/2$, ${\bar{y}}_{P1}$ is the EMM for one parent, ${\bar{y}}_{P2}$ is the EMM for the other parent, and ${\bar{y}}_{F1}$ is the EMM for the hybrid. BPH ratios were estimated by $({\bar{y}}_{F1}-{\bar{y}}_{BP})/{\bar{y}}_{BP}$, where ${\bar{y}}_{BP}$ is the EMM for the best-parent. The best parent for an elite×elite hybrid (blue circles) was the parent with the largest EMM, whereas the best parent for an elite × exotic hybrid (red circles) was the elite parent. The shades of blue identify hybrids (n=356) within different elite×elite full-sib families, whereas the shades of red identify hybrids (n=113) within different elite × exotic full-sib families. The solid blank lines are predicted values from linear regressions of BPH ratio on MPH ratio.

**Fig. 5.**
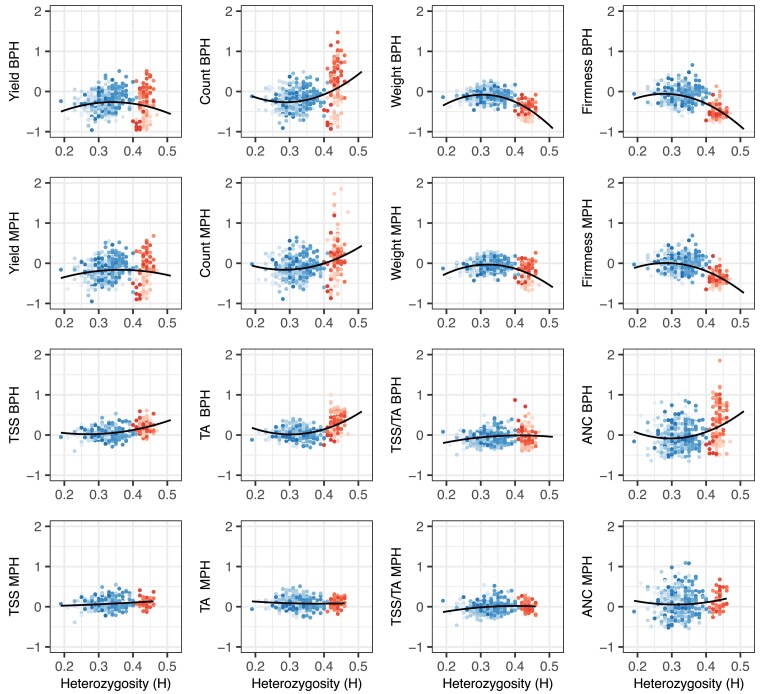
Heterozygosity (H) by mid-parent heterorsis (MPH) and best-parent heterosis (BPH) ratios for fruit yield, count, weight, firmness, TSS, TA, TSS/TA, and ANC among 469 strawberry hybrids grown in Salinas, California field experiments in 2017–2018 and 2018–2019. H was estimated from a genome-wide sample of 28,513 Axiom 50K array-genotyped SNPs. MPH ratios were estimated by $({\bar{y}}_{F1}-{\bar{y}}_{MP})/{\bar{y}}_{MP}$, where ${\bar{y}}_{MP}=({\bar{y}}_{P1}+{\bar{y}}_{P2})/2$, ${\bar{y}}_{P1}$ is the EMM for one parent, ${\bar{y}}_{P2}$ is the EMM for the other parent, and ${\bar{y}}_{F1}$ is the EMM for the hybrid. BPH ratios were estimated by $({\bar{y}}_{F1}-{\bar{y}}_{BP})/{\bar{y}}_{BP}$, where ${\bar{y}}_{BP}$ is the EMM for the best-parent. The best parent for an elite×elite hybrid (blue points) was the parent with the largest EMM, whereas the best parent for an elite × exotic hybrid (red points) was the elite parent. The shades of blue identify hybrids within different elite×elite full-sib families (n=356), whereas the shades of red identify hybrids within different elite × exotic full-sib families (n=113). The solid blank lines are predicted values from linear or quadratic regressions of BPH ratios on *H*.

**Fig. 6.**
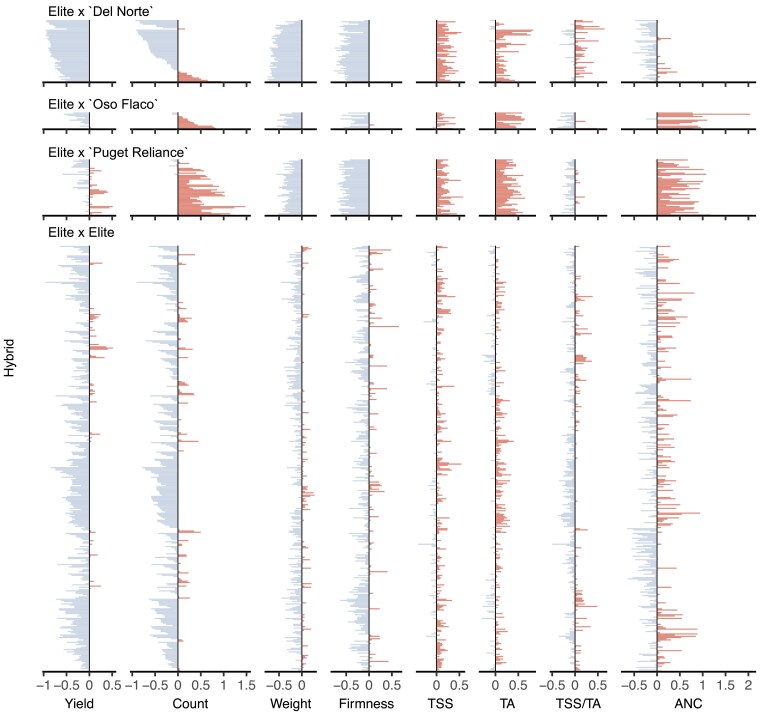
Best-parent heterosis (BPH) ratios for fruit yield, count, weight, firmness, total soluble solids (TSS), TA, TSS/TA, and ANC among 469 strawberry hybrids grown in Salinas, California field experiments in 2017–2018 and 2018–2019, where $({\bar{y}}_{F1}-{\bar{y}}_{BP})/{\bar{y}}_{BP}$ in the BPH ratio, ${\bar{y}}_{BP}$ is the estimated marginal mean (EMM) for the best parent, and ${\bar{y}}_{F1}$ is the EMM for the hybrid. Contrasts between hybrid and best-parent EMMs ($\text{BPH}={\bar{y}}_{F1}-{\bar{y}}_{BP}$) were estimated for every best parent–hybrid combination. The best parent for an elite×elite hybrids was the parent with the largest EMM, whereas the best parent for an elite × exotic hybrids was the elite parent. The exotic parents were “Puget Reliance”, “Oso Flaco”, and “Del Norte”. BPH ratios in the negative range are shown in gray, whereas BPH ratios in the positive range are shown in red.

Our breeding history and genome-wide association studies suggested that the between-parent allele frequency differences and dispersed favorable dominant alleles necessary for heterosis have diminished within the California population (Lamkey and Edwards 1999; Birchler *et al*. 2003, 2010; Kaeppler 2012; Schnable and Springer 2013; Mackay *et al*. 2021). To explore this, we estimated mid-parent heterosis ($\text{MPH}={\bar{y}}_{F1}-{\bar{y}}_{MP}$) and best-parent heterosis ($\text{BPH}={\bar{y}}_{F1}-{\bar{y}}_{BP})$ among hybrids developed with and without exotic parents, where ${\bar{y}}_{BP}$ is the EMM for the best-parent, ${\bar{y}}_{MP}=({\bar{y}}_{P1}+{\bar{y}}_{P2})/2$ is the mid-parent mean, ${\bar{y}}_{P1}$ is the EMM for one parent, ${\bar{y}}_{P2}$ is the EMM for the other parent, and ${\bar{y}}_{F1}$ is the EMM for the hybrid (Figs. 4–6; Supplementary File 1). When both parents of the hybrid were elite, the best-parent was identified as the parent with the greater mean (${\bar{y}}_{BP}=\max[{\bar{y}}_{P1},{\bar{y}}_{P2}])$. When one parent was exotic, the elite parent was identified as the best parent because we sought to identify exotic parents that were sources of novel (dispersed) favorable alleles for improving the performance of elite×elite hybrids (Meredith and Bridge 1972; Dudley 1982, 1987; Schnable and Springer 2013; Bernardo and Thompson 2016; Mayer *et al*. 2020; Mackay *et al*. 2021).

MPH and BPH were strongly positively correlated for every trait (Fig. 4). The correlations were strongest for fruit yield, weight, firmness, TSS, and TSS/TA (0.93–0.97) and slightly weaker for fruit count, TA, and ANC (0.75–0.86). The lower correlation for fruit count (0.76) was apparently caused by differences in the strengths, frequencies, and distributions of favorable alleles transmitted by the exotic parents of hybrids within elite × Puget Reliance and elite × Del Norte heterotic patterns (Figs. 4 and 6). The lower MPH × BPH correlations for TA (0.75) and ANC (0.86) were apparently caused by differences between elite × Puget Reliance and elite×elite hybrids (Fig. 4). MPH could not be estimated for hybrids within the elite × Oso Flaco heterotic pattern because the exotic parent (Oso Flaco) lacked stolons and could not be clonally propagated (or phenotyped). Similarly, MPH could not be estimated for TSS, TA, or ANC among elite × Del Norte hybrids because clones of the wild parent (Del Norte) either produced zero or one to two small fruit each. BPH, however, was estimated for every hybrid in each heterotic pattern and shed light on the prevalence and effects of favorable dominant alleles transmitted by elite and exotic parents alike (Fig. 6).

Hybrids in the elite×elite heterotic group had the highest yields and largest and firmest fruit and dominated the apexes of the positive parabolic H × EMM distributions for fruit yield and weight (the tranche of elite×elite hybrids with heterozygosities in the 0.27≤H≤0.40 range depicted by blue points in Fig. 2). The median heterozygosity for elite×elite hybrids from the upper 10% of the phenotypic distribution for fruit yield was $\tilde{y}=0.33$. We found that 94.4 to 98.6% of elite×elite hybrids had BPH estimates that were either significantly less than zero (${\bar{y}}_{F1}<{\bar{y}}_{BP}$) or not significantly different from zero (${\bar{y}}_{F1}={\bar{y}}_{BP}$) for fruit yield, count, weight, and firmness (Table 2; Figs. 5 and 6). The percentage of statistically significant contrasts that were negative (${\bar{y}}_{F1}<{\bar{y}}_{BP}$) for these traits ranged from 33.1 to 50.6% among elite×elite hybrids (Table 2; Figs. 5 and 6). Hence, best-parent heterosis (${\bar{y}}_{F1}>{\bar{y}}_{BP}$) was scarce among elite×elite hybrids for traits improved by direct selection (fruit yield, weight, and firmness) within the CA population (Fig. 6).

**Table 2.** The percentage of elite×elite and elite×exotic hybrids with mid-parent heterosis (MPH) and best-parent heterosis (BPH) contrast estimates that were significantly negative, not significantly different, or significantly positive, where $\text{MPH}={\bar{y}}_{F_{1}}-{\bar{y}}_{MP}$, $\text{BPH}={\bar{y}}_{F_{1}}-{\bar{y}}_{BP}$, ${\bar{y}}_{F_{1}}$ is the EMM for the hybrid, ${\bar{y}}_{MP}=({\bar{y}}_{P1}+{\bar{y}}_{P2})/2$ is the mid-parent EMM, ${\bar{y}}_{P1}$ is the EMM for one parent, ${\bar{y}}_{P2}$ is the EMM for the other parent, and ${\bar{y}}_{BP}$ is the EMM for the best parent.

	Elite×Elite (%)				Elite×Exotic (%)			
Trait	*n*	${\bar{y}}_{F_{1}}<{\bar{y}}_{MP}$	${\bar{y}}_{F_{1}}={\bar{y}}_{MP}$	${\bar{y}}_{F_{1}}>{\bar{y}}_{MP}$	*n*	${\bar{y}}_{F_{1}}<{\bar{y}}_{MP}$	${\bar{y}}_{F_{1}}={\bar{y}}_{MP}$	${\bar{y}}_{F_{1}}>{\bar{y}}_{MP}$
Yield (g/plant)	356	26.7	71.1	2.2	99	43.4	48.5	8.1
Count (fruit)	356	23.9	70.8	5.3	99	22.2	46.5	31.3
Weight (g/fruit)	356	12.9	83.1	3.9	99	56.6	42.4	1.0
Firmness (kg-force)	356	12.9	78.9	8.1	99	85.9	14.1	0.0
TSS (%)	356	0.0	99.7	0.3	47	0.0	100.0	0.0
TA (%)	356	2.2	78.7	19.1	47	0.0	76.6	23.4
TSS/TA	356	4.5	89.9	5.6	47	0.0	95.7	4.3
ANC (*μ*g/mL)	356	5.1	77.8	17.1	47	0.0	76.6	23.4

	Elite × Elite (%)				Elite × Exotic (%)			
Trait	*n*	${\bar{y}}_{F_{1}}<{\bar{y}}_{BP}$	${\bar{y}}_{F_{1}}={\bar{y}}_{BP}$	${\bar{y}}_{F_{1}}>{\bar{y}}_{BP}$	*n*	${\bar{y}}_{F_{1}}<{\bar{y}}_{BP}$	${\bar{y}}_{F_{1}}={\bar{y}}_{BP}$	${\bar{y}}_{F_{1}}>{\bar{y}}_{BP}$
Yield (g/plant)	356	50.6	48.0	1.4	113	58.4	36.3	5.3
Count (fruit)	356	49.2	48.0	2.8	113	31.9	23.0	45.1
Weight (g/fruit)	356	28.4	68.5	3.1	113	96.5	3.5	0.0
Firmness (kg-force)	356	33.1	61.2	5.6	113	95.6	4.4	0.0
TSS (%)	356	0.8	93.0	6.2	113	0.0	64.6	35.4
TA (%)	356	6.2	82.9	11.0	113	0.9	31.0	68.1
TSS/TA	356	13.8	82.3	3.9	113	21.2	61.9	16.8
ANC (*μ*g/mL)	356	14.9	74.4	10.7	113	8.0	55.8	36.3

EMMs were estimated from phenotypes of parents and hybrids observed across clonal replicates, harvests, and years for fruit yield, count, weight, firmness, TSS, TA, and ANC. The best parent for an elite×elite hybrid was the parent with the largest EMM, whereas the best parent for an elite × exotic hybrid was the elite parent. The percentages shown were calculated from tests of the null hypothesis of no MPH ($H_{0}$: ${\bar{y}}_{F_{1}}-{\bar{y}}_{MP}=0$) or no BPH ($H_{0}$: ${\bar{y}}_{F_{1}}-{\bar{y}}_{BP}=0$) for every hybrid using a 5% Type I error threshold.

The best-parent heterosis uncovered for fruit yield among elite×elite hybrids was not only scarce but limited to the widest hybrids among them, primarily between “Camarosa” and elite parents that emerged 20–23 years later (Figs. 4 and 5; Table 3; Supplementary File 1). MPH for fruit yield was significant for only eight elite×elite hybrids, six of which had “Camarosa” as a common parent (Supplementary File 1). BPH was significant for five of those hybrids, four of which had “Camarosa” as a common parent. “Camarosa”, the oldest and one of the most genetically divergent elite parents used in our study, emerged in 1988 shortly before a founder effect bottlenecked the CA population (Pincot *et al*. 2021). The best-parent heterosis uncovered for fruit weight was similarly scarce among elite×elite hybrids and uncorrelated with BPH for fruit yield (Table 2; Fig. 6; Supplementary File 1). BPH was statistically significant for fruit weight for 13 elite×elite hybrids among 11 different parents, none of which were “Camarosa”. Our findings suggest that dispersed dominant favorable alleles are uncommon for fruit yield and weight among modern descendants of the California population but not completely absent and not necessarily shared by the same individuals for different traits (Kaeppler 2012; Mayer *et al*. 2020; Mackay *et al*. 2021). We concluded that baseline heterosis, the heterosis lost as a consequence of inbreeding (Lamkey and Edwards 1999; Labroo *et al*. 2021), could not be restored by hybridization of individuals within the CA population despite the residual heterozygosity carried in their genomes (Table 1; Fig. 2; Supplementary File 1).

**Table 3.** The percentage of elite×elite hybrids (n=356) and elite × exotic (elite × “Puget Reliance”) hybrids (n=47) with phenotypic means that were less than the worst parent (${\bar{y}}_{F_{1}}<{\bar{y}}_{WP}$), where ${\bar{y}}_{F_{1}}$ is the EMM of the hybrid and ${\bar{y}}_{WP}$ is the EMM of the worst parent.

	Elite × Elite (%)		Elite × Exotic (%)	
Trait	${\bar{y}}_{F_{1}}<{\bar{y}}_{WP}$	P<0.05	${\bar{y}}_{F_{1}}<{\bar{y}}_{WP}$	P<0.05
Yield (g/plant)	54.2	9.3	10.6	0.0
Count (fruit)	48.6	13.5	12.8	0.0
Weight (g/fruit)	45.2	5.6	0.0	0.0
Firmness (kg-force)	41.9	2.2	31.9	0.0
TSS (%)	15.1	0.0	0.4	0.0
TA (%)	13.2	0.8	0.0	0.0
TSS/TA	36.8	1.1	23.4	0.0
ANC (*μ*g/mL)	26.9	0.0	0.4	0.0

The percentage of statistically significant worst-parent contrasts (p<0.05) was estimated from tests of the null hypothesis of no difference between hybrid and worst-parent EMMs ($H_{0}$: ${\bar{y}}_{F_{1}}-{\bar{y}}_{WP}=0$) using a 5% Type I error threshold. EMMs were estimated from phenotypes of parents and hybrids observed across clonal replicates, harvests, and years for fruit yield, count, weight, firmness, TSS, TA, and ANC.

### Wide hybrids uncover significant heterosis for fruit count, a critical driver of genetic gains for fruit yield

Heterosis for fruit count appears to have been a significant driver of genetic gains for fruit yield in strawberry (Figs. 4–6; Table 3; Supplementary File 1). Even though fruit count was not directly selected, genetic gains for fruit yield could not have been achieved in the CA population without a significant increase in fruit count (Feldmann *et al*. 2024). Similar to what we observed for fruit yield, heterosis for fruit count was uncommon among elite×elite hybrids but not completely lacking (Table 2; Figs. 4–6; Supplementary File 1). Although the additive genetic correlation between fruit yield and count was strongly positive among elite×elite hybrids ($r_{G}=0.84$), best-parent heterosis (${\bar{y}}_{F1}>{\bar{y}}_{BP}$) was only statistically significant for both traits for a single elite×elite hybrid (“Camarosa” × 07C092P003; Supplementary File 1). We concluded that the allele frequency differences necessary for heterosis have decreased within the CA population as a consequence of a breeding-associated increase in inbreeding (Lamkey and Edwards 1999; Kaeppler 2012; Mackay *et al*. 2021).

BPH ratios for fruit count ranged from 0.26 to 0.49 with a median of $\tilde{y}=0.35$ among the 10 elite×elite hybrids with statistically significant BPH contrasts (Table 2; Fig. 6). Of those 10 hybrids, seven had either “Camarosa” or “Portola” as a common parent (Supplementary File 1). That result was instructive because “Canarosa” and “Portola” are more closely related to early CA population and non-CA individuals than the other elite parents and hybrids tested in our study (Fig. 1; Supplementary File 1). BPH ratios for fruit yield and count were strongly correlated (r=0.859;P<0.0001) among elite×elite hybrids and had virtually identical distributions (Fig. 6). This implies that direct selection for increased fruit yield and weight targeted favorable dominant alleles that simultaneously increased fruit count (Figs. 3–6). We concluded that directional dominance among fruit count-associated loci drove genetic gains for fruit yield and that favorable dominant alleles for fruit yield, count, and weight have simultaneously increased in frequency and homozygosity within the CA population despite the slightly negative additive genetic correlation between fruit count and weight ($r_{G}=-0.26$) among elite×elite hybrids (Fig. 3). The additive genetic correlation between fruit yield and weight was notably less positive among elite×elite hybrids ($r_{G}=0.26$) than elite × exotic hybrids ($r_{G}=0.77$). This difference was undoubtedly partly caused by significant differences in allele frequencies between these hybrid groups (Figs. 2 and 3). Our genome-wide association studies suggest that additive genetic variation for fruit weight has been nearly eliminated and that many fruit-weight associated favorable alleles are either homozygous or approaching homozygosity within the CA population (Fig. 3).

Significant heterosis was observed for fruit count among elite × exotic hybrids, especially those developed with Puget Reliance and Oso FLaco (Table 2; Figs. 4–6; Supplementary File 1). BPH contrasts for fruit count were significantly positive (${\bar{y}}_{F1}>{\bar{y}}_{BP}$) for 45.1% of elite × exotic hybrids (percent BPH ranged from 25 to 147% with a median of 52%) and significantly negative (${\bar{y}}_{F1}<{\bar{y}}_{BP}$) for 31.9% of elite × exotic hybrids. The wild relative (Del Norte) was the exotic parent for 100.0% of the latter. Conversely, 97.2% of the BPH contrasts for fruit count were either significantly negative (${\bar{y}}_{F1}<{\bar{y}}_{BP}$) or not significantly different from zero (${\bar{y}}_{F1}={\bar{y}}_{BP}$) among elite×elite hybrids, which suggests that favorable alleles for fruit count have been driven to fixation or near fixation within the CA population (Table 2; Supplementary File 1). These findings are aligned with the finding that 100% of the BPH contrast estimates for fruit weight were negative among elite × exotic hybrids, and show that favorable alleles for fruit count transmitted by the exotic parents were unfavorable for fruit weight (Table 2–3; Fig. 6; Supplementary File 1).

Simultaneous direct selection for increased fruit yield and weight greatly increased fruit count within the CA population despite a slightly negative genetic correlation between fruit weight and count ($r_{G}=-0.26$) among elite×elite hybrids. Interestingly, the genetic correlation between fruit count and weight was close to zero among elite × exotic hybrids ($r_{G}=0.09$). Elite × exotic hybrids from the upper 20% of the fruit count distribution had fruit weights in the 6.91 to 24.6 g/fruit range with a median of $\tilde{y}=17.3$, whereas elite×elite hybrids from the upper 20% of the fruit count distribution had fruit weights in the 17.5 to 35.1 g/fruit range with a median of $\tilde{y}=24.9$, the approximate lower limit necessary for large-scale production (Fig. 2; Supplementary File 1). These results suggest that the pleiotropic effects of favorable alleles for fruit count introduced by founders of the CA population did not prevent the simultaneous improvement of fruit yield and weight, even though a preponderance of the favorable alleles for fruit count found in those founders and other exotic sources are predicted to decrease fruit weight (Tables 2 and 3; Fig. 6).

### Hybrids with exotic parents exposed the pleiotropic effects of loci targeted by selection within the California population

Our companion study of historical genetic gains within the California population showed that TSS and TA were indirectly selected and decreased over cycles of direct selection for increased fruit yield, weight, and firmness in the California population (Feldmann *et al*. 2024). We attributed those genetic losses (TSS and TA decreases) to breeding for high yields of large, long shelf life fruit, and more specifically to the pleiotropic effects of loci targeted by direct selection for increased fruit yield, weight, and firmness. Our MPH and BPH estimates support that conclusion and suggest that the alleles transmitted by the exotic parents were predominantly unfavorable for fruit weight and firmness and favorable for TSS and TA (Figs. 2–5).

BPH contrasts for fruit weight and firmness were mirror images of those for TSS and TA within the three elite × exotic heterotic patterns (Fig. 6). BPH contrasts (${\bar{y}}_{F1}-{\bar{y}}_{BP}$) were negative for 99.1–100.0% of elite × exotic hybrids for fruit weight and firmness (the directly selected traits) and positive for 89.4–92.9% of elite × exotic hybrids for TSS and TA (the indirectly selected traits). The percentage of elite × exotic hybrids with significant best-parent heterosis (${\bar{y}}_{F1}>{\bar{y}}_{BP}$) was 35.4% for TSS and 68.1% for TA (Table 2). Conversely, the percentage of elite × exotic hybrids with significant negative contrasts (${\bar{y}}_{F1}<{\bar{y}}_{BP}$) was 95.6% for fruit firmness and 96.5% for fruit firmness (Table 2). MPH was significant for 19.1% of elite×elite hybrids for TA as opposed to 0.3% for TSS; hence, allelic variation for TA-associated loci appears to be greater than that for TSS-associated loci within the CA population (Table 2), a hypothesis supported by our genome-wide association study (Fig. 3).

The BPH ratio distributions for hybrids within the three elite × exotic heterotic patterns shared predictable similarities and noteworthy differences (Fig. 6). First, heterosis for fruit yield was limited to the elite × Puget Reliance heterotic pattern among the three elite × exotic heterotic patterns. BPH ratios for fruit yield were negative for every hybrid within the elite × Oso Flaco and elite × Del Norte heterotic patterns (Fig. 6). Interestingly, the maximum BPH ratios for hybrids within the elite × Puget Reliance and elite×elite heterotic patterns were identical (0.51). Those hybrids were Puget Reliance × 11C180P001 and Camarosa × 07C092P003 (Supplementary File 1). Moreover, the nine hybrids from the upper 2% of the fruit yield BPH ratio distributions for these heterotic patterns had either Puget Reliance or Camarosa as a common parent (Fig. 1).

Second, heterosis for fruit count was observed within each elite × exotic heterotic pattern, but differed in frequency and strength among them (Fig. 6). BPH ratios for fruit count were positive for nearly every elite × Oso Flaco and elite × Puget Reliance hybrid and negative for 79.2% of elite × Del Norte hybrids. Heterosis was stronger and the BPH ratio range was wider for fruit count for hybrids with “Puget Reliance” (−0.20 to 1.47 with a median of $\tilde{y}=0.49$) than “Oso Flaco” (−0.13 to 0.83 with a median of $\tilde{y}=0.25$). This suggested that Puget Reliance, which is more genetically distant to the CA population than Oso Flaco (Fig. 1), transmitted favorable alleles that were larger in number or greater in effect than those transmitted by Oso Flaco, or a combination thereof.

Third, the wild parent (Del Norte) primarily transmitted unfavorable alleles for fruit count (86.0% of the BPH contrasts for elite × Del Norte hybrids were negative), a pattern strikingly different from that observed for Puget Reliance and Oso Flaco (Fig. 5; Table 2; Supplementary File 1). We concluded that Puget Reliance and Oso Flaco have accumulated favorable alleles for fruit count that are not present in Del Norte (the wild parent). Nevertheless, the three exotic parents transmitted favorable alleles for fruit count that are either not present or have largely been fixed or lost within the CA population (Figs. 3 and 6).

### The fixation of favorable dominant alleles theoretically explains the fixation of heterosis within the California population

Our findings suggest that heterosis for fruit yield, count, and weight was greater in early, less inbred, and more genetically diverse generations of the CA population, equivalent to the heterosis observed for other traits in our study (Figs. 5–6; Table 2), before declining in later generations as the between-parent allele frequency differences necessary for heterosis decreased (Lamkey and Edwards 1999; Birchler *et al*. 2010; Kaeppler 2012; Birchler 2013; Mackay *et al*. 2021). We hypothesize that as that happened (as favorable dominant alleles increased in frequency and many became fixed), heterosis for fruit yield, count, and weight disappeared, as predicted by generation means theory for a single locus with two alleles, where ${\text{lim}}_{\;p\to 1}f(p)=0$, p=1−q is the frequency of the favorable dominant allele, *q* is the frequency of the unfavorable recessive allele, and f(p) is the generation mean for a particular mating design (Lamkey and Edwards 1999). The theoretical generation means for inbred-MPH, panmictic-MPH, and baseline heterosis are solely functions of *d* (the dominance deviation) and (p−q)/2 (half the allele frequency difference) for a single locus with two alleles (Lamkey and Edwards 1999).

Our argument is that heterosis progressively declined in the CA population as frequencies of favorable dominant alleles progressively increased (as p→1) and inbreeding increased across large swaths of the genomes of modern descendants of the California population (Ritland 1984; Hardigan *et al*. 2021). Of the 10 hybrids in our study with ¿ 30% best-parent heterosis for fruit yield, six were elite × “Puget Reliance” (the only non-CA population cultivar tested), and four were elite × “Camarosa”, the oldest and most genetically distant CA population cultivar tested (Fig. 1; Supplementary File 2). Percent BPH ranged from 30 to 50% among those 10 hybrids, which suggests that a deeper exploration of genetically divergent elite and exotic donors should uncover significant heterosis for fruit yield in strawberry and might identify sources of favorable alleles that are not present among the parents of elite hybrids to be improved (Dudley 1982, 1984; Kaeppler 2012; Mayer *et al*. 2020; White *et al*. 2020; Mackay *et al*. 2021).

### The prevalence of heterosis for an unselected trait supports the hypothesis of heterosis fixation for selected traits

The H × BPH distribution for ANC, a trait that does not appear to have ever been under direct or indirect selection in the CA population (Feldmann *et al*. 2024), differed from that observed for traits that were (Figs. 5 and 6). ANC was genetically uncorrelated with fruit yield among elite×elite hybrids in our study (${\hat{r}}_{G}=0.00$). The additive effects of ANC-associated genetic variants were similar in our genome-wide association studies of hybrids developed with and without exotic parents, which sharply contrasted with what we observed for fruit weight, firmness, TSS, and TA (Fig. 3). This implied that allelic variation among ANC-associated loci was comparable among hybrids developed with and without exotic parents and that allelic variation has persisted for ANC-associated loci within the CA population. This conclusion was supported by the segregation of large-effect ANC-associated loci on chromosomes 1C and 6A and nearly identical percentages of significant MPH contrasts, and comparable additive effect estimate distributions, for ANC among elite×elite and exotic × exotic hybrids (Table 2; Figs. 3–6). Our genetic analyses of those large-effect loci are to be described in a companion paper.

BPH contrast estimates for ANC were statistically significant for 10.7% of elite×elite and 36.3% of elite × exotic hybrids (Fig. 6; Table 2). Percent BPH for ANC ranged from 36 to 101% among 39 elite×elite and from 38 to 184% among 40 elite × exotic hybrids with statistically significant BPH contrasts (Supplementary File 1). In sharp contrast to what we observed for fruit firmness, TSS, and TA, elite and exotic parents appear to be sources of favorable alleles for modifying ANC in either direction (Table 2; Figs. 3–6). These findings suggest that heterosis may have been more widespread and significant for traits under direct or indirect positive selection (fruit yield, count, weight, and firmness) in early generations of the CA population, a conclusion supported by the prevalence of significant BPH for fruit yield among elite × Puget Reliance hybrids and fruit count among elite × Puget Reliance and elite × Oso Flaco hybrids and remnants of BPH for fruit yield and count among elite×elite hybrids with “Camarosa” or “Portola” as parents (Figs. 1–6; Table 2).

### The restoration of heterosis in wide hybrids further supports the hypothesis of heterosis fixation

Our GWAS findings suggest that favorable alleles have accumulated nearly genome-wide among loci affecting fruit yield, count, weight, and firmness within the California population and that the between-parent allele frequency differences present in the early founders have largely disappeared in the high yielding, large-fruited modern descendants we analyzed (Fig. 3). This hypothesis was supported by the high frequency of negative and scarcity of positive transgressive segregates for those traits among elite×elite hybrids (Tables 2 and 3; Supplementary File 1).

The phenotypic means for 85% of the elite×elite hybrids were less than the best parent for fruit yield (Table 3; Supplementary File 1). Moreover, nearly half of the elite×elite hybrids in our study had phenotypic means for fruit yield, count, weight, and firmness that were less than the worst parent (Table 3). The percentages of these contrasts (${\bar{y}}_{F_{1}}-{\bar{y}}_{WP}$) that were negative and statistically significant (P<0.05) among elite×elite hybrids ranged from 2.2% for fruit firmness to 13.5% for fruit count, where ${\bar{y}}_{WP}$ is the phenotypic mean of the worst parent (Table 3; Supplementary File 1). The same percentages for fruit quality traits ranged from 0.0% for TSS and ANC to 0.8% for TA among elite×elite hybrids. Strikingly, we did not observe a single elite × “Puget Reliance” hybrid with a phenotypic mean that was significantly less than the worst parent (Table 3). These results are perfectly aligned with our inbreeding depression and heterosis results (Tables 1 and 2; Figs. 4–6), and suggest that alleles segregating in the noninbred fractions of the genomes of the elite parents are predominantly unfavorable for traits that were directly selected (fruit yield, weight, and firmness) or indirectly selected (fruit count) and positively genetically correlated with fruit yield in the California population (Feldmann *et al*. 2024).

The highest yielding strawberry cultivars reported to-date are amongst the most highly inbred (Figs. 2–5; Hardigan *et al*. 2021; Feldmann *et al*. 2024). This seems counterintuitive, especially for a highly heterozygous, outbreeding, interspecific hybrid species harboring heavy genetic loads (Table 1; East 1934, 1936; Shaw 1995, 1997). What are the implications of long-term selection within intentionally bottlenecked strawberry populations designed to deliver elite genetics, as exemplified by the CA population? Such populations become more inbred, and as they do (and as the CA population has), the individuals within them lose the allele frequency differences necessary for heterosis (Falconer and Mackay 1996; Lamkey and Edwards 1999; Kaeppler 2012). Generation-means models of heterosis for a single locus with two alleles for different pedigrees and mating designs (e.g. inbred-MPH, panmictic-MPH, and baseline heterosis) are functions of allele frequencies and the dominance deviation (Lamkey and Edwards 1999). Our analyses suggest that heterosis disappeared in the California population (e.g. MPH →0) as favorable alleles approached fixation (p→1) among loci targeted by selection for improved hybrid performance (Figs. 2–6).

Hybrid breeding strategies are designed to maximize hybrid performance not heterosis *per se* (Melchinger and Gumber 1998; Duvick 2005; Troyer 2006; Schnable and Springer 2013), and here California population hybrids excel (Feldmann *et al*. 2024). While high performing hybrids are genetically distant (by definition), genetic distance is generally a poor predictor of hybrid performance, e.g. see Flint-Garcia *et al*. (2009). This hybrid breeding axiom stresses that hybrid performance and heterosis are often correlated but not synonymous (Lamkey and Edwards 1999; Kaeppler 2011; Schnable and Springer 2013). The hypothesized fixation (disappearance) of heterosis within the California population should not be interpreted as a negative outcome of breeding but rather as the natural and inevitable consequence of greatly improved hybrid performance driven by the accumulation of favorable and elimination of unfavorable alleles within that population (Figs. 2–3). The dominance hypothesis predicts the decline of heterosis as the frequencies of favorable dominant alleles approach fixation between parents, parent groups, or populations (Lamkey and Edwards 1999; Kaeppler 2012; Schnable and Springer 2013; Labroo *et al*. 2021). Our hypothesis is that the heterosis inherent in wide hybrids and diverse populations of the hybrid species dissipated and ultimately disappeared within the California population as the homozygosity of dominant favorable alleles for fruit yield, count, weight, and firmness increased (Feldmann *et al*. 2024). This conclusion is supported by the finding that heterosis for fruit yield and count was restored among elite × “Puget Reliance” hybrids (Figs. 4–6; Supplementary File 1).

Our findings suggest that complementation of unfavorable recessive alleles by dominant favorable alleles (as predicted by the dominance hypothesis) could be the prevalent driver of heterosis in strawberry (Charcosset and Essioux 1994; Vuylsteke *et al*. 2000; Birchler *et al*. 2003, 2006; Springer and Stupar 2007; Birchler *et al*. 2010; Schnable and Springer 2013). We are proposing this in part to explain the balance that seems to have been achieved between the avoidance of inbreeding depression and the inevitable decay of heterosis in the CA population where selection for hybrid performance appears to have greatly increased the homozygosity of favorable dominant alleles and consanguineous matings and selective sweeps have naturally and inexorably increased inbreeding (Smith and Haigh 1974; Fay and Wu 2000; Wright and Gaut 2005). If a preponderance of those favorable alleles were dominant, and heterosis was largely caused by favorable dominant alleles masking the effects of deleterious recessive alleles, theory predicts that the phenotypes of favorable allele homozygotes and heterozygotes should be nearly identical and that heterosis should disappear because of the fixation of favorable dominant alleles (Lamkey and Edwards 1999; Birchler *et al*. 2006, 2010; Kaeppler 2012; Schnable and Springer 2013). This is precisely what we suspect our hybrids show (Figs. 2–6).

The erosion of allelic variation that arises from truncation selection in closed populations predictably affects the maintenance of genetic variation for selected and unselected traits, partly because of genetic drift and selection at linked sites (Prezeworski *et al*. 2005; Chen *et al*. 2010; Stephan 2019; Khoury *et al*. 2022; Cole 2023). The inbreeding observed in the CA population has simultaneously decreased allelic diversity, increased homozygosity, and driven genetic gains for fruit yield, count, and weight (Fig. 3; Feldmann *et al*. 2024), heterotic traits that are depressed by inbreeding (Table 2; Lynch 1991; Shaw 1995; Shaw and Larson 2008; Charlesworth and Willis 2009). How then has the exposure of the genetic load by inbreeding (inbreeding depression) been offset by the greatly increased vigor and yields of modern hybrids originating in the CA population? Our hypothesis is that modern breeding has purged a significant fraction of the genetic load (deleterious alleles) found in wild relatives, heirloom cultivars, and founders of the CA population and driven favorable dominant alleles for specific traits to near fixation or fixation.

The inbreeding observed in the CA population has been good from the standpoint of improving traits critical for large-scale production (fruit yield, weight, and firmness), presumably by increasing the frequencies and homozygosities of partially to completely dominant favorable alleles, whilst avoiding or counteracting the depression associated with increased homozygosity of deleterious incompletely dominant alleles (Lynch 1991; Charlesworth and Willis 2009; Yang *et al*. 2017; Lozano *et al*. 2021; Feldmann *et al*. 2024). With 97,000 to 108,000 genes packed into 28 chromosomes (Edger *et al*. 2019; Jin *et al*. 2023; https://phytozome-next.jgi.doe.gov/info/FxananassaRoyalRoyce_v1_0), eliminating those unfavorable alleles poses a daunting challenge that entails breaking repulsion linkages among massive numbers of linked loci and identifying and recovering favorable allele recombinants in coupling phase along entire chromosome arms (Bailey and Comstock 1976; Gorelick and Laubichler 2004; Wallace *et al*. 2018; Dietrich *et al*. 2021). This is one of the most prevalent and daunting challenges ahead for modern breeding (Schnable and Springer 2013; Yang *et al*. 2017).

### Good inbreeding and inbreeding depression avoidance in heterotic, outbreeding species

The effects of inbreeding in animal and plant populations are often detrimental, but differ as a function of life history, mating systems, and other factors (Lande and Schemske 1985; Charlesworth and Willis 2009). Lynch (1991) noted that “with almost no exception, empirical studies indicate that inbreeding causes a shift in mean phenotypes in a direction that causes a reduction in fitness”. The problem, however, is not as simple as concluding that all “inbreeding is bad”, especially in domesticated populations where deleterious alleles have been purged and favorable alleles have accumulated over many generations of direct selection and centuries of domestication (Lynch 1991; Charlesworth and Willis 2009; Colli *et al*. 2018; Maltecca *et al*. 2020; Lozada-Soto *et al*. 2021; Cole 2023).

The improvement of maize inbred lines within heterotic groups is a well know example of good inbreeding in a heterotic outbreeder, one that fundamentally changed breeding schemes and hybrid seed production practices (Duvick 2001). The yields of maize inbred lines have been improved by line *per se* selection within heterotic groups since the 1930s without affecting percent heterosis (Russell 1991; Duvick 1984, 1999, 2001; Troyer 2006; Birchler *et al*. 2010; Kaeppler 2012; Schnable and Springer 2013), presumably by eliminating incompletely dominant deleterious alleles (Gore *et al*. 2009; Yang *et al*. 2017; Mayer *et al*. 2020; White *et al*. 2020; Lozano *et al*. 2021). The prevailing hypothesis is that different complements of such alleles have persisted in the genomes of maize inbred lines within heterotic groups, especially in pericentromeric and other genomic regions where recombination is suppressed (Melchinger 1999; Gore *et al*. 2009; Yang *et al*. 2017; Mayer *et al*. 2020; White *et al*. 2020; Lozano *et al*. 2021). Our findings suggest that the good inbreeding observed in the California strawberry population has arisen in a way similar to that observed for maize inbred lines within heterotic groups (Russell 1991; Duvick 2001; Troyer 2006), albeit more slowly by selection among hybrid offspring from consanguineous hybrid × hybrid crosses rather than by line *per se* selection among inbred (e.g. self-pollinated or doubled-haploid) offspring from within-heterotic group inbred line × inbred line crosses (Fig. 2). The important distinction here is that between group hybrids still greatly outperform within group inbreds in maize.

Similar to strawberry, inbreeding has progressively increased in many cattle breeds (closed populations), presumably by achieving a delicate balance between eliminating deleterious alleles and accumulating favorable alleles whilst avoiding inbreeding depression (Maltecca *et al*. 2020; Lozada-Soto *et al*. 2021; Cole 2023). Strawberry, at least the closed population we studied, appears to have been down nearly the same path as cattle and other domesticated animals (Varona *et al*. 2016; Xiang *et al*. 2016; Lozada-Soto *et al*. 2021; Cole 2023), albeit with important distinctions. First, inferior progeny are simply discarded in strawberry by applying intense truncation selection where the focus is the identification and advancement of an exceedingly small number of outstanding hybrid individuals. Second, because of the first distinction, gene flow between populations is not only less of a barrier in strawberry and other domesticated plants than domesticated animals (Varona *et al*. 2016; Xiang *et al*. 2016), but commonly used to introduce novel favorable alleles into closed populations to solve breeding problems and purposefully introduce allelic variation (Tanksley and McCouch 1997; Zamir 2001; Schouten *et al*. 2019; Jiménez *et al*. 2022; Pincot *et al*. 2022; Feldmann *et al*. 2023; Knapp *et al*. 2023; McCouch and Rieseberg 2023).

The heterosis of maize hybrids has been widely attributed to the complementation of incompletely dominant deleterious alleles found in contrasting heterotic groups (Melchinger and Gumber 1998; Melchinger 1999; Gore *et al*. 2009; Kaeppler 2012; van Heerwaarden *et al*. 2012; Schnable and Springer 2013; Yang *et al*. 2017; Mayer *et al*. 2020; Lozano *et al*. 2021). Such masking can obviously only occur in the noninbred fractions of the genomes of hybrids within moderately to highly inbred strawberry populations (e.g. the CA population), which nevertheless segregate for the equivalent of a diploid genome or more and harbor significant loads of incompletely dominant deleterious alleles despite historical inbreeding (Table 1). Interestingly, the absence of BPH and statistically significant positive transgressive segregation suggests that baseline heterosis (the heterosis lost by inbreeding the noninbred fractions of the genomes of California population individuals) was not restored among elite×elite hybrids (Fig. 5; Tables 1 and 2). This suggests that the complement of loci in the noninbred fractions contributes substantially less to hybrid performance than complement of loci fixed in the inbred fractions.

Selection in domesticated plants and animals leads to the accumulation of linked favorable alleles for multiple traits across entire chromosomes and genomes (Laurie *et al*. 2004; Technow *et al*. 2014; Colli *et al*. 2018; Frantz *et al*. 2020; Dietrich *et al*. 2021; Cole 2023), albeit with nonuniform distributions (Gore *et al*. 2009; Albrecht *et al*. 2014; Yang *et al*. 2017; Lozano *et al*. 2021). The co-adapted gene complexes that arise could be caused by epistasis or linkage disequilibrium or both among the causal loci (Lynch 1991; Gorelick and Laubichler 2004; Dietrich *et al*. 2021; Mackay *et al*. 2021). The frequencies and arrays of favorable alleles that differ and diverge between populations, e.g. between breeds, market classes, and heterotic groups, can be extreme, particularly in plant species where inbred-hybrid breeding schemes have been applied (Gore *et al*. 2009; van Heerwaarden *et al*. 2012; Windhausen *et al*. 2012; Albrecht *et al*. 2014; Yang *et al*. 2017; Mayer *et al*. 2020). Given the scarcity of favorable alleles for TSS and TA among elite parents, ubiquity of favorable alleles for TSS and TA among exotic parents, and pronounced nearly genome-wide differences in genetic variants associated with these traits among hybrids developed with and without exotic parents (Fig. 3), we concluded that the exotic parents disrupted haploblocks of favorable alleles for fruit yield, count, weight, and firmness that have accumulated, mostly in coupling, across the genomes of the elite parents (Dietrich *et al*. 2021; Mackay *et al*. 2021). There are no shortages of favorable alleles for TSS and TA among the diverse and highly admixed genetic backgrounds of North American and European cultivars, both elite and exotic (Gil-Ariza *et al*. 2009; Horvath *et al*. 2011; Lerceteau-Köhler *et al*. 2012; Chambers *et al*. 2014; Sánchez-Sevilla *et al*. 2015; Vallarino *et al*. 2018; Hardigan *et al*. 2021; Pincot *et al*. 2021; Fan *et al*. 2022); however, most are likely to have negative pleiotropic effects on fruit yield, weight, and firmness (Figs. 2–6).

### Improving hybrid performance in asexually propagated plants without exploiting heterotic groups and patterns

Although heterotic groups and patterns were never developed or needed to achieve significant genetic gains in strawberry (Feldmann *et al*. 2024), the standing genetic variation needed to create and exploit heterotic groups and introduce favorable dominant alleles into elite heterotic groups from exotic donors clearly exists (Figs. 4–6). Our findings suggest that heirloom cultivars and other non-CA populations harbor favorable dominant alleles that are not found in the CA population (Figs. 2–5). Strawberry populations with independent breeding histories that have genetically diverged (Gil-Ariza *et al*. 2009; Horvath *et al*. 2011; Sánchez-Sevilla *et al*. 2015; Vallarino *et al*. 2018; Hardigan *et al*. 2021) undoubtedly harbor complementary favorable dominant alleles that could be transferred and exploited between populations and used to create heterotic groups and patterns analogous to those created in maize (Dudley 1984, 1987; Melchinger and Gumber 1998; van Heerwaarden *et al*. 2012; Mayer *et al*. 2020).

The within-population breeding schemes applied in strawberry are somewhat similar to the within-breed breeding schemes applied in domesticated animals (Maltecca *et al*. 2020; Lozada-Soto *et al*. 2021; Cole 2023), and markedly different from the between heterotic group breeding schemes applied in maize that have been so important for informing our understanding of heterosis and hybrid breeding strategies in plants (Duvick 2001; Troyer 2006; Birchler *et al*. 2010; Kaeppler 2012; Schnable and Springer 2013; Labroo *et al*. 2021). With maize, heterotic groups were initially formed *ad hoc* and genetically diverged over nearly a century of selection for improved testcross hybrid (between-heterotic group) performance (Melchinger and Gumber 1998; Melchinger 1999; Reif *et al*. 2005; van Heerwaarden *et al*. 2012; Albrecht *et al*. 2014; Technow *et al*. 2014). With strawberry, selection for improved hybrid performance has been applied to individuals either within highly unstructured (admixed) or highly structured and closed populations (Hancock *et al*. 2008; Whitaker *et al*. 2012; Hardigan *et al*. 2021; Feldmann *et al*. 2024).

The weakly to highly unstructured and admixed populations found in strawberry (Gil-Ariza *et al*. 2009; Horvath *et al*. 2011; Sánchez-Sevilla *et al*. 2015; Vallarino *et al*. 2018; Hardigan *et al*. 2021) are less inbred than the CA population and somewhat analogous to the open-pollinated populations of maize selected as sources of individuals for creating heterotic groups in the 1930s (Melchinger and Gumber 1998; Duvick 2001, 2005; Reif *et al*. 2005; Troyer 2006; van Heerwaarden *et al*. 2012). Coincidentally, the formation of maize heterotic groups occurred about the same time that strawberry breeding got underway at the University of California (Hancock 2006; Pincot *et al*. 2021; Feldmann *et al*. 2024) and that East (1934, 1936) was carrying out his groundbreaking work on heterosis in maize and other species of plants, including strawberry. The breeding of maize and strawberry, despite the inbreeding depression and heterosis known to be significant in both species, understandably went down completely different paths from the 1930s onward, primarily because of differences in breeding mechanics and plant propagation practices (East 1934, 1936; Darrow 1966; Sjulin and Dale 1987; Duvick 2001; Troyer 2006; Hancock *et al*. 2008; van Heerwaarden *et al*. 2012; Hardigan *et al*. 2021).

Why go down the path of forming heterotic groups and patterns in strawberry and other asexually propagated heterotic species where they have not been formed, a strategic decision that invariably increases resource needs and necessitates the application of inbred-hybrid breeding schemes? The utilization of such schemes has been limited in strawberry because hybrid individuals can be asexually propagated, thus exploiting 100% of the additive and nonadditive genetic variation among individuals and circumventing the need for laborious, time-consuming, and expensive inbred line and hybrid development and testing schemes (Lynch and Walsh 1998; Bernardo 2002; Labroo *et al*. 2021). Within-population breeding schemes require substantially fewer resources than inbred-hybrid breeding schemes (Labroo *et al*. 2021) without sacrificing hybrid performance, as shown in our strawberry example (Fig. 2; Feldmann *et al*. 2024). The latter should be true in any species where directional dominance is the predominant genetic mechanism underlying heterosis (Lamkey and Edwards 1999; Birchler *et al*. 2003, 2010; Kaeppler 2011; Schnable and Springer 2013; Labroo *et al*. 2021).

As shown in our companion study, genetic gains from the long-term application of heterosis-unaware within population selection have been substantial in strawberry (Feldmann *et al*. 2024). Whether or not fruit yields can be further increased by exploiting heterotic groups and patterns and applying inbred-hybrid breeding schemes remains unclear. Our study predictably showed that heterotic groups and patterns can be created between genetically divergent strawberry populations for the development of seed-propagated single-cross hybrids using inbred-hybrid breeding schemes (Fig. 1). The latter have been proposed as a means for protecting intellectual property and circumventing disease problems caused by soil-borne pathogens that plague asexual propagation, even if they are completely unnecessary for maximizing hybrid performance and replicating the uniformity of asexually propagated hybrid individuals (Labroo *et al*. 2021; Feldmann *et al*. 2024).

## Data Availability

The raw genotypic and phenotypic data and Supplementary files associated with our studies are available in a DRYAD repository: https://doi.org/10.5061/dryad.866t1g20j. These include Supplementary Fig. 1 and Supplementary Files 1–8.
